# Profiling Novel, Multifunctional Silane-Phosphonate Consolidants for the Mitigation of Gypsum Stone Deterioration via Concerted Autocondensation/Surface Complexation Processes

**DOI:** 10.1021/acs.cgd.4c00327

**Published:** 2024-07-08

**Authors:** Stefania Liakaki-Stavropoulou, Argyri Moschona, Ioannis E. Grammatikakis, Duane Choquesillo-Lazarte, Konstantinos D. Demadis

**Affiliations:** † Crystal Engineering, Growth and Design Laboratory, Department of Chemistry, University of Crete, Voutes Campus, Heraklion GR-71003, Crete, Greece; ‡ Laboratorio de Estudios Cristalográficos, IACT, CSIC-Universidad de Granada, Granada 18100, Spain

## Abstract

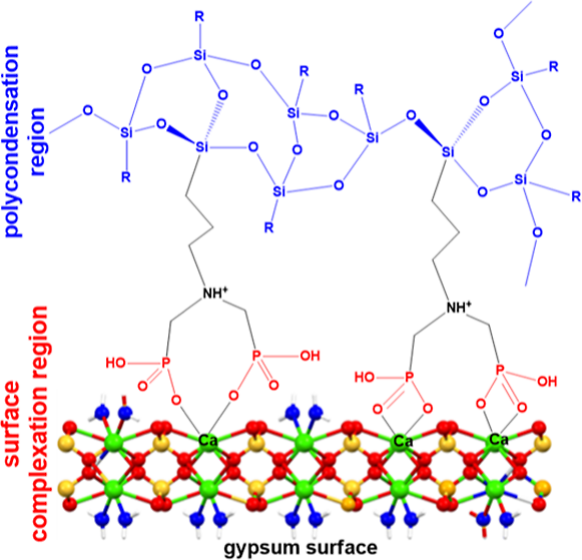

Mineral gypsum (selenite) stones have been used extensively by ancient Cretans in the Minoan Palace of Knossos (Crete, Greece), mostly for building and ornamental purposes. Exposure of mineral gypsum to environmental stresses (temperature fluctuations, rain, air-borne pollutants, soluble salts, etc.) causes solubility-driven degradation, and loss of cohesion of the crystal aggregates, with ensuing aesthetic degradation. In this work, the efficiency of four consolidants for artificial gypsum specimens is presented and evaluated based on drilling resistance measurements [drilling resistance measuring system (DRMS)]. Two of them (commercial names RC-70 and RC-90, RC = Rhodorsil Consolidante) are alkoxysilane-based and they are considered as benchmark consolidants. The other two [3-(trihydroxysilyl)propyl methylphosphonate monosodium salt, TRIMEPHONA, and 3-(trihydroxysilyl)propylamino-diphosphonate, TRIPADIPHOS] are multifunctional consolidants because they possess a self-condensable (after hydrolysis) trihydroxysilyl [−Si(OH)_3_] moiety and phosphonate groups (one in the former, two in the latter). Consolidants RC-70 and RC-90 exhibit rather low consolidation effectiveness. This is not unexpected, as these are alkoxysilane-based and act simply as “fillers” for the pores of the gypsum. Consolidant TRIMEPHONA demonstrates an enhanced level of consolidation action. This is due to its double functionality, i.e., the presence of an anionic phosphorus-based moiety that anchors onto the gypsum surface, and a condensable silane triol [−Si(OH)_3_] unit. Consolidant TRIPADIPHOS shows excellent gypsum consolidation features and is much more efficient (per unit concentration) than all other tested consolidants. This is assigned to its better gypsum anchoring ability via surface Ca-complexation. Selected compressive strength studies were performed on gypsum samples treated with the phosphorus-based consolidants, and corroborate the findings from DRMS. To shed further light on possible binding modes of the phosphonate moiety on surface Ca^2+^ sites in gypsum, two model compounds were synthesized and structurally characterized, Ca–C2D and Ca–C3D (C2D = ethylamino-di(methylenephosphonic acid) and C3D = propylamino-di(methylenephosphonic acid).

## Abstract 2

The multifunctional consolidant 3-(trihydroxysilyl)propylamino-diphosphonate, TRIPADIPHOS, demonstrates an enhanced level of consolidation action on gypsum. This is ascribed to its double functionality, i.e., the presence of two anionic phosphonate moieties that anchor onto the gypsum surface, and a condensable silane triol [−Si(OH)_3_] unit that generates an extended covalent network within the gypsum pores.

## Introduction

Mineral gypsum (selenite) stones were used extensively by ancient Cretans in the Minoan Palace of Knossos (Crete, Greece), mostly for building and ornamental purposes. Interestingly, the mineral gypsum building and ornamental elements catalogue of the palatial complex of Knossos consists of 2185 entries.^1^ However, from a chemical standpoint, Knossian gypsum presents two significant problematic issues: its rather high water-solubility (compared to other minerals used for construction purposes, such as marble) and its softness. Consequently, exposure of mineral gypsum to environmental stresses (temperature fluctuations, rain, air-borne pollutants, soluble salts, etc.) causes gradual dissolution, and loss of cohesion of the crystal aggregates, with ensuing aesthetic degradation. One of the main issues concerning the state of preservation of the Knossian mineral gypsum (selenite) is its solubility, given that gypsum is a rather soft and water-soluble mineral, with a *K*_sp_ of ∼3.14 × 10^–5^.^2^ The dissolution of gypsum leads to loss of cohesion between the crystalline aggregates and, eventually, to degradation and loss of original material.

Gypsum in all its forms is a rather “soft” mineral (hardness value of 2 on the Mohs scale) and is profoundly affected by environmental factors and the conditions to which it is exposed.^3^ The decay mechanisms, usually triggered by environmental factors, are principally related to its chemical properties. Specifically, its solubility exerts a serious impact that is enhanced in the presence of increasing concentrations of soluble salts.^4,5^ The decay is manifested as decohesion of the gypsum crystalline aggregates and erosion and microcarst formations, mostly in the form of runnels.^3,6^ The macroscopic characteristics of the aforementioned phenomena notwithstanding, the common factor is the loss of original material, eventually leading to the degradation of this unique, and therefore of crucially important, aspect of the Palace of Knossos.

Mineral gypsum is a challenging material from a conservation point of view because its translucency (one of the most significant aspects of mineral gypsum) can be easily compromised. For example, an attempt was based on the transformation of the decayed calcium sulfate dihydrate crystalline aggregates into barium sulfate with limited success.^7^ This was due to the fact that the more insoluble barite that is produced is opaque, thus leading to visual alteration of the original appearance of mineral gypsum. Within the framework of mineral gypsum conservation and preservation, several commercially available products with known properties (advantages and disadvantages) designated for stone consolidation, have been tested.^8,9^ The common feature of their modus operandi is their ability to form transparent final products via a sol–gel process. Given that translucency is one of the most desirable aesthetic values of mineral gypsum to be preserved, this property is of crucial importance.

Following up on the above considerations, artificial gypsum samples were prepared in the laboratory. Because such samples can be prepared easily and are available at an unlimited supply, all experiments were done with this type of gypsum. The efficacy of the consolidants was assessed in terms of enhancement of the mechanical properties of gypsum, brought about by the consolidants’ action. Among other techniques, this can be achieved through the application of drilling resistance measurements [drilling resistance measuring system (DRMS)]. DRMS has been used as an evaluation tool in several cases, where the efficacy of consolidation treatments was in question.^10−12^ In general, the DRMS results show a correlation with the porosity and its fluctuations before and after consolidation treatments.^13,14^ Importantly, DRMS was used as a high precision sampling tool, collecting the drilling residue (dust) from specific interval depths. The drilling residue was then analyzed with several other analytical techniques. Hence, useful information on the state of preservation of the successive strata of the evaluated stone, and the performance of the consolidants can be extracted.

**Figure 1:**
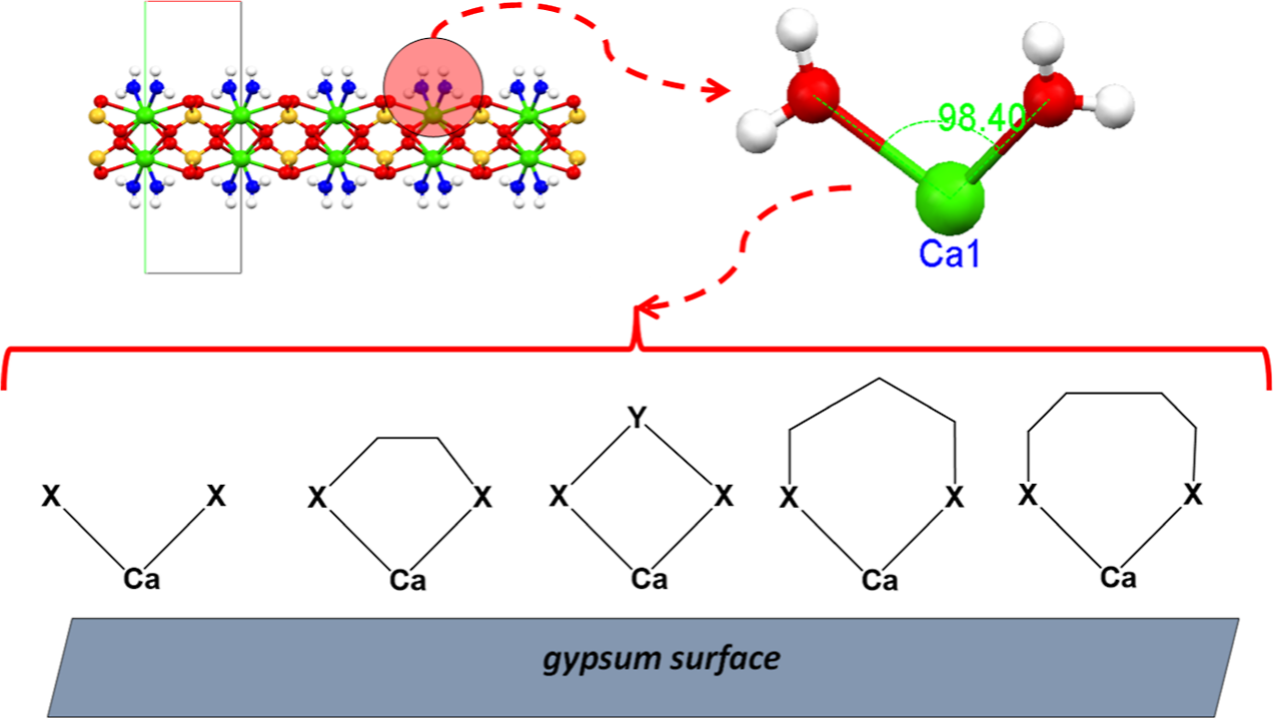
Consolidant anchoring strategy onto a gypsum surface. The “X” symbol denotes either water or anionic groups on the consolidant molecule. Color codes: calcium, green; oxygen, red (the Ca-bound waters are differentiated in blue in the gypsum layer image); hydrogen white; sulfur, yellow.

In this paper, selected organic (TRIMEPHONA and TRIPADIPHOS) and inorganic (alkoxysilane-based) chemical consolidants have been tested on artificial gypsum samples. The inorganic consolidants (RC-70 and RC-90) are commercial and were used in the study for comparison purposes, as a “baseline”. To the best of our knowledge, none of the above chemical compounds have been tested on gypsum. RC-70 and RC-90 were evaluated before on various stone materials,^15^ e.g., marly limestones.^16^ In all cases the main parameter was the compatibility of the consolidating material with the sulfate substrate. However, for the organic consolidants the working hypothesis was that the anionic phosphonate moieties on TRIMEPHONA and TRIPADIPHOS could potentially substitute the water molecules bound to the Ca^2+^ ions “exposed” to the interlayer space in the structure of gypsum. Such an approach takes advantage of the high affinity of the anionic phosphonate group (R-PO_3_^2–^) for calcium, either in solution,^17^ or on a surface.^18^ This anchoring strategy is outlined in Figure 1. After the coordination of consolidant onto the gypsum surface, the silane triol [−Si(OH)_3_] portion of the molecule can undergo polycondensation reactions with neighboring molecules, thus creating a Si–O–Si network, similar to that created by hydrolyzed tetraethoxysilane (TEOS).

## Experimental Section

### Materials

#### Gypsum Samples

Gypsum belongs to the family marine evaporites, originally precipitated from a saturated surface or near-surface brine dried by solar evaporation.^19^ It is composed of calcium cations (Ca^2+^) and sulfate anions (SO_4_^2–^) and two molecules of water (bound to the cation Ca^2+^), with the chemical formula CaSO_4_·2H_2_O per unit. The crystal structure of gypsum is monoclinic, with 2/m symmetry, with four molecules per unit cell.^20^ It demonstrates successive layers (sheets) of calcium sulfate “molecules” with the calcium-coordinated water molecules facing toward the intralayer region. The layers are composed of strongly ionic Ca–O (sulfate) bonds that are distributed within each layer. However, the interlayer interactions are based on much weaker hydrogen bonds.^21^ Therefore, the Mohs scale of mineral hardness defines gypsum as hardness value 2 (based on scratch hardness comparison) and as a phyllitic mineral. Moreover, the calcium sulfate/water layers are arranged parallel to the (010) crystallographic plane, a fact that is reflected on the perfect cleavage along this plane.^22^

The main goal of this investigation was focused on the documentation and evaluation of consolidation efficiency of selected multifunctional chemical compounds on artificial gypsum samples. Given the limited quantity (if any) of the original mineral material from the archeological site of Knossos (selenite) and keeping in mind that the final objective of this approach is related mostly to the chemical bonding (surface coordination chemistry) of the consolidating compounds with the surface of gypsum, artificial samples were prepared and used along with the mineral gypsum samples. For the molding of the artificial gypsum samples Knauf Rocaso (EN 13279-1) was used with a hemihydrate purity of 95%. Hemihydrate was dispersed over laboratory deionized water through a sieve with aperture size of 0.85 mm (ASTM: 20). According to the manufacturer, the final product has the following characteristics: flexural strength ≥3.0 N/mm^2^, compressive strength ≥5.0 N/mm^2^ and dry density ∼1 g/cm^3^.

#### Alkoxysilane-Based Consolidants

**Figure 2:**
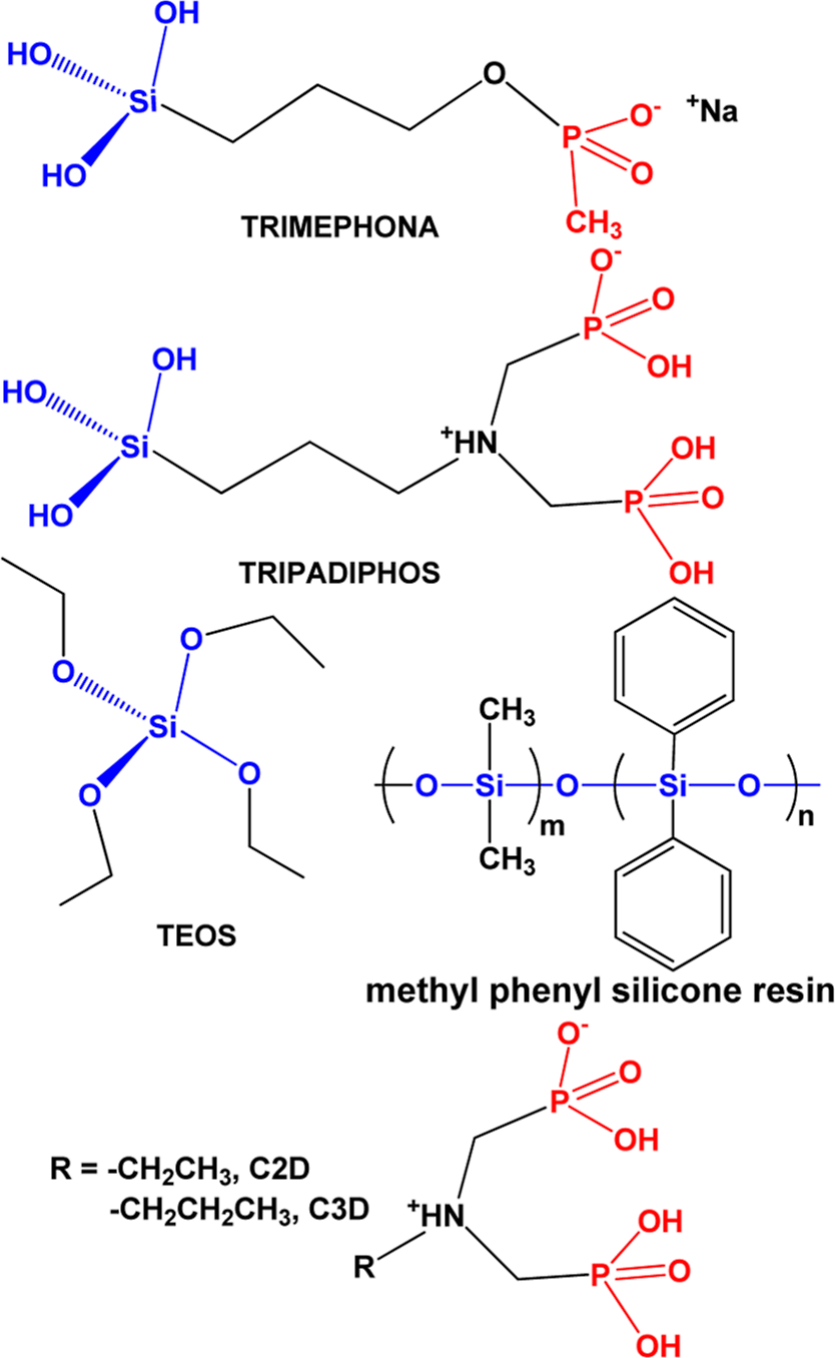
Schematic structures of the two multifunctional consolidants (TRIMEPHONA and TRIPADIPHOS) and of the components in the commercial alkoxysilane consolidants RC-70 (TEOS) and RC-90 (TEOS + methylphenyl silicone resin) evaluated in this study, with their abbreviations. The structures of the two “model” phosphonates (C2D and C3D) are also given.

The alkoxysilanes and alkyl alkoxysilanes, or “silanes” in short, RC-70 and RC-90 (RC stands for Rhodorsil consolidante) are used in this work as “benchmark” consolidation materials, so appropriate comparisons can be drawn with the multifunctional consolidants (see below). The former have been extensively used as stone consolidants for a long time.^9^ Their observed consolidating performance is due to their ability to form Si–O–Si bonds with the substrate, thus significantly improving the mechanical properties of stone.^8^ However, this is feasible only for silicate stones and also for limestones, containing certain amounts of silica.^23^ Despite the fact that gypsum is not a silicate-bearing salt, alkoxysilanes have been used as reference consolidants in relevant studies.^14^ In particular, the TEOS based consolidants are dominant. The two commercially available products based on ethyl silicates were (see Figure 2): (a) Rhodorsil RC-70 (TEOS 30 v/v % in white spirit) and (b) Rhodorsil RC-90 (TEOS with methylphenylsilicone resin 24%/6 v/v %, in white spirit and toluene). The silicon content was approximately 70%, the viscosity at 25 °C was ∼1 mm/s and the aliphatic hydrocarbons content was <50%. The cross-linking reaction was initiated and accelerated by dibutyltin dilaurate (DBTL, R_2_Sn(OOCR′)_2_) where R = −(CH_2_)_3_CH_3_ and R′ = −(CH_2_)_10_CH_3_. The choice of the aforementioned materials is based on the type of solvent used in the polycondensation reaction of TEOS, as well as on the catalyst (DBTL) since they are directly related to the aggregation pathway followed by the growing particles in the sol–gel transition.^24^

#### Multifunctional Consolidants

Two multifunctional consolidants were evaluated, see Figure 2. The first is 3-(trihydroxysilyl)propyl methylphosphonate monosodium salt, TRIMEPHONA, is a commercial product (obtained from Aldrich, CAS Number: 84962-98-1, as a 50% w/v aqueous solution). 3-(Trihydroxysilyl)propylamino-diphosphonate (TRIPADIPHOS) was synthesized via the Mannich-type methodology (Figure S1 in the Supporting Information).^25^ Based on this synthetic strategy an aliphatic primary amine can be converted to an amino-di(methylenephosphonate) [−N(CH_2_PO_3_H_2_)_2_] moiety. Specifically, in a 20 mL glass round-bottom flask charged with a reflux condenser, the following were added: 5 mL deionized water, 5.534 g (0.025 mol) (3-aminopropyl)triethoxysilane (APTES, purchased from Alfa-AESAR), 4.1 g (0.05 mol) phosphorus acid and 5 mL HCl (37% w/v). The mixture was brought to the boiling point and was kept there for 1 h. Subsequently, 7.5 mL formaldehyde were added dropwise for 30 min, and the mixture was kept under heating for 3 h. Then, the condenser was removed, and the water was removed under constant heating to yield an orangish precipitate, which was isolated by vacuum filtration. The isolated solid was washed repeatedly with methanol to remove any colored byproducts. TRIPADIPHOS was characterized by ^1^H NMR, ^13^C NMR, ^29^Si NMR and ATR-IR spectroscopies. These data can be found in the Supporting Information (Figures S2–S5). The solid was also analyzed by Electron Dispersive Spectrometry (EDS, see Figure S6 in the Supporting Information), which confirmed the expected Si/P atom ratio of 1:2. TRIPADIPHOS is a water-soluble compound and can be used to prepare aqueous solutions of appropriate concentrations, as needed. However, above ∼10 mM self-condensation processes start to occur, and as a result the functionality of the −Si(OH)_3_ group is compromised.

#### Model Phosphonate Compounds

The aminomethylenephosphonic acids ethylamino-di(methylene phosphonic acid) (C2D) and propylamino-di(methylene phosphonic acid) (C3D), Figure 2, were synthesized via the Mannich-type methodology (Figure S1 in the Supporting Information).^25^

#### Synthesis of Ca–C2D

0.2 mmol (0.044 g) of C2D was dissolved in 2 mL of DI water and the pH was adjusted at 2 by using stock solution NaOH 1 M. An equimolar quantity of CaCl_2_·2H_2_O salt (0.2 mmol, 0.029 g) was added to the above solution under stirring. The final mixture was placed in a Teflon-lined reactor and was heated at 120 °C for 3 days. After cooling at room temperature, the clear solution was left at room temperature and after 3 days colorless crystals formed and were isolated by filtration and washed with small quantity of DI water. Yield 11%, based on the metal salt.

#### Synthesis of Ca–C3D

0.4 mmol (0.98 g) of C3D was dissolved in 2 mL of DI water and the pH was adjusted at 4.5 by using stock solution NaOH 1M. 0.2 mmol (0.029 g) of CaCl_2_·2H_2_O salt was added to the above solution under stirring. The final mixture was placed in a Teflon-lined reactor and was heated at 140 °C for 4 days. After cooling at room temperature, colorless crystals formed and were isolated by filtration and washed with small quantity of DI water. Yield 6% based on the metal salt.

### Methods

#### Preparation of Artificial Gypsum Specimens

**Figure 3:**
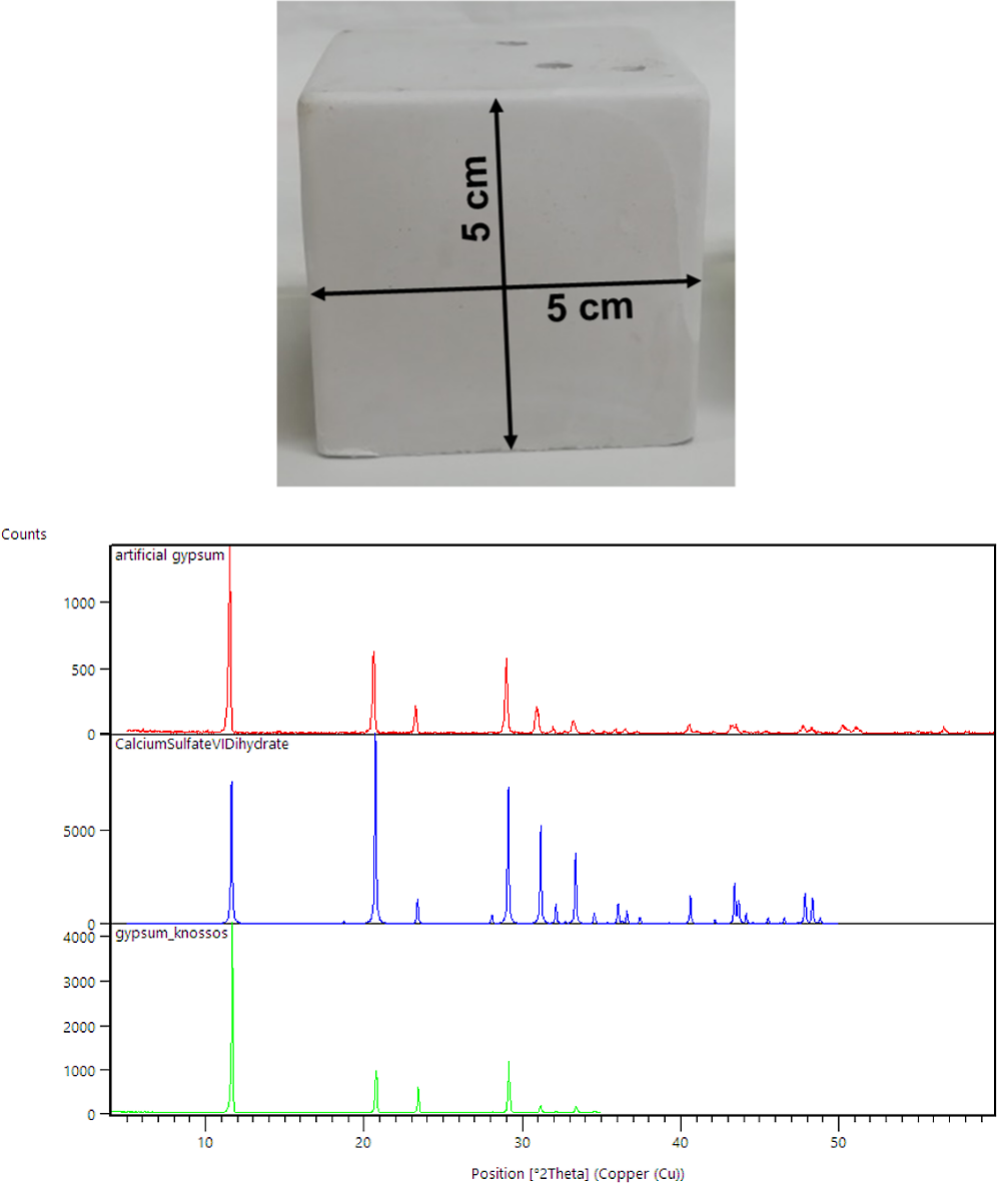
(Upper) representative specimen of artificial gypsum. All samples of artificial gypsum are of external dimensions 5 × 5 × 5 cm^3^. (Lower) agreement between calculated (blue), experimental (artificial laboratory-made, red) and mineral (from Knossos, green) gypsum XRD powder pattern diagrams.

Calcium sulfate hemihydrate (CaSO_4_·1/2H_2_O, “plaster of Paris”) was used as the precursor. Hence, 1 kg was added to 600 mL of deionized water and the mixture was mixed gently with a plastic spatula to avoid formation of bubbles until it became a homogeneous greyish thick/viscous fluid. The freshly prepared material was poured into silicone molds of cubic shape (dimensions 5 × 5 × 5 cm^3^) within 10 min, where it was left to settle and solidify at ambient conditions. After 2 h, the semidry specimens were removed from the molds and were left exposed to air for further drying for at least 1 week. At the end of this process all calcium sulfate hemihydrate has been transformed to calcium sulfate dihydrate (CaSO_4_·2H_2_O, gypsum). Complete transformation was ensured by powder XRD (Figure 3), which showed peaks corresponding to gypsum exclusively. Figure 3 also shows the morphological features of the gypsum specimens.

#### Consolidation Treatment

All gypsum samples were immersed in Petri dishes (9 cm base diameter) containing 30 mL consolidant solution (up to ∼1 cm height). The samples were divided into two groups, and each group underwent one and two consecutive immersions, respectively. Each cycle consisted of 60 min immersion and for the second group of samples, 24 h intermission between immersions. After the consolidant absorption process is complete, the specimen is allowed to dry under ambient conditions for 28 days (for consolidants RC-70 and RC-90, per supplier instructions) or 7 days (for consolidants TRIMEPHONA and TRIPADIPHOS). Oven drying must be avoided because it can cause gypsum dehydration. Consolidants RC-70, RC-90 and TRIMEPHONA were used as received (see above), without any dilution. Solid TRIPADIPHOS was used to prepare aqueous solutions of concentrations 3.33 and 10 mM.

### Instrumentation

The efficiency evaluation was based on the drilling resistance measured in Newton (N) and the tool applied for the acquisition of the data was DRMS by Sint Technology, Italy. The apparatus was equipped with 5 mm Diaber–Sint drill bit and a guide hole, operating at a rotational speed (ω) = 600 rpm with and penetration rate (υ) = 10 mm/min. The average drilling depth was 50 mm. The drillings were done on the gypsum surface that was not initially immersed in the consolidant solution and as close as possible to the center of the cube side. The data points presented are averages of three drilling measurements (in some cases more) and the error margin is ∼±10%.

Furthermore, the micromorphology, as well as the chemical composition of the final products in terms of molecular, elemental, and stoichiometric analysis was examined by means of scanning electron microscopy coupled with energy dispersing X-ray spectrometry (EDS) and attenuated total reflectance (ATR) infra-red (IR) (ATR-IR) spectroscopic analyses. The samples examined through this analytical protocol consisted of the drilling residue that was produced during the drilling resistance measurement of the gypsum samples. The micromorphological observations as well as the elemental qualitative and quantitative analyses were performed with a Jeol JSM 6390LV scanning electron microscope (SEM) with analytical working distance of 10 mm, with a spatial resolution of 3.0 nm (@30 kV), equipped with an embedded energy dispersive X-ray analyzer (EDS). The ATR-IR spectra were recorded on a Thermo Fisher Scientific Nicolet iS10 FTIR spectrometer operating in ATR mode with a spectral range between 7800 and 350 cm^–1^ and spectral resolution ∼0.4 cm^–1^. Compressive strength measurements were performed on samples of artificial gypsum of external dimensions 5 × 5 × 5 cm^3^ (prepared as above). Three types of samples (groups of 4) were measured: (a) control (no consolidant present), (b) those treated with TRIMEPHONA, and (c) those treated with TRIPADIPHOS. The tests were performed on a computer-controlled E161-03N servo plus type instrument from MATEST S.p.A. (Bergamo, Italy) with dual range 500/15 kN and a loading rate of 0.500 MPa/s. The apparatus includes parallel loading plates for transferring the load to the specimen and a spherical bearing head on top of the specimen, the axis of which must be aligned with the axis of the specimen and the center of the loading plate.

### X-ray Crystallography

X-ray diffraction data were collected at room temperature from a single-crystal mounted atop a glass fiber under Paratone-N oil, with a Bruker SMART APEX II diffractometer using graphite-monochromated Mo–Kα (λ = 0.71073 Å) radiation. The data were processed with the APEX3 suite.^26^ The structures of Ca–C2D and Ca–C3D were solved by intrinsic phasing using the ShelXT program,^27^ which revealed the position of all non-hydrogen atoms. These atoms were refined on F^2^ by a full-matrix least-squares procedure, using the anisotropic displacement parameter.^28^ All hydrogen atoms were located in difference Fourier maps and included as fixed contributions riding on attached atoms with isotropic thermal displacement parameters 1.2- or 1.5-times those of the respective atom. The Olex2 software was used as a graphical interface.^29^ Molecular graphics were generated using Mercury.^30^ The structures have been deposited with the CCDC, with the following code numbers: 2332950 (for Ca–C2D) and 2253212 (for Ca–C3D).

## Results and Discussion

This work focuses on the evaluation of consolidation efficiency (i.e., the ability of a chemical compound to provide cohesion to the stone of gypsum) based on the utilization of a DRMS. It demonstrated how this test can provide information on the penetration depth of the consolidant as well as the penetration resistance variations.^31,32^ DRMS can at the same time be used as a high precision sampling tool, because the drilling residue (dust) can be collected from specific interval depths and then analyzed with several other analytical techniques (e.g., powder X-ray diffraction, SEM, etc.). Hence, useful information regarding both the state of preservation of the successive strata of the examined rock as well as the performance of the consolidants can be extracted.

## Performance of Multifunctional Consolidants

**Figure 4:**
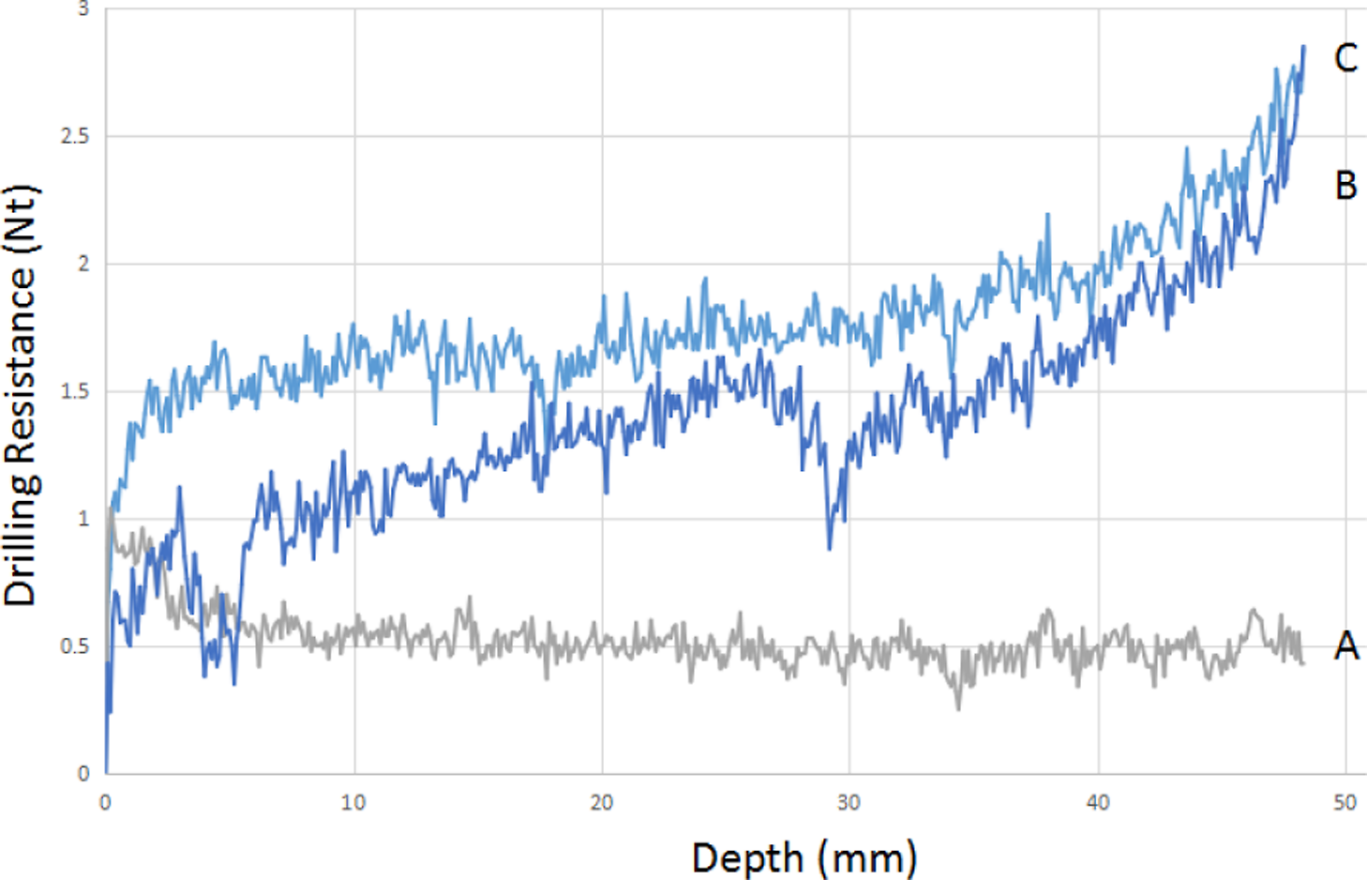
Evaluation of TRIMEPHONA solution application on artificial gypsum via DRMS. (A) corresponds to the control (untreated), (B) to the first immersion, and (C) to the second immersion.

In the case of gypsum samples treated with an aqueous TRIMEPHONA solution (50% w/v), a significant increase in drilling resistance has been documented, see Figure 4. The initial drilling resistance of untreated gypsum (trace A: 0.67 N) was increased to 1.45 N (trace B, 116% increase) and 1.78 N (trace C, 166% increase) after one and two immersions, respectively. The “spikes” observed are ascribed to the sample inhomogeneities and imperfections and are always present in all samples tested. The drilling resistance seems to improve with penetration depth, and is an indication that the consolidant has penetrated to a depth of up to 50 mm. This is desirable from a practical standpoint, as surface (or close-to-surface) condolidation is not adequate and offers only superficial stone cohesion.

**Figure 5:**
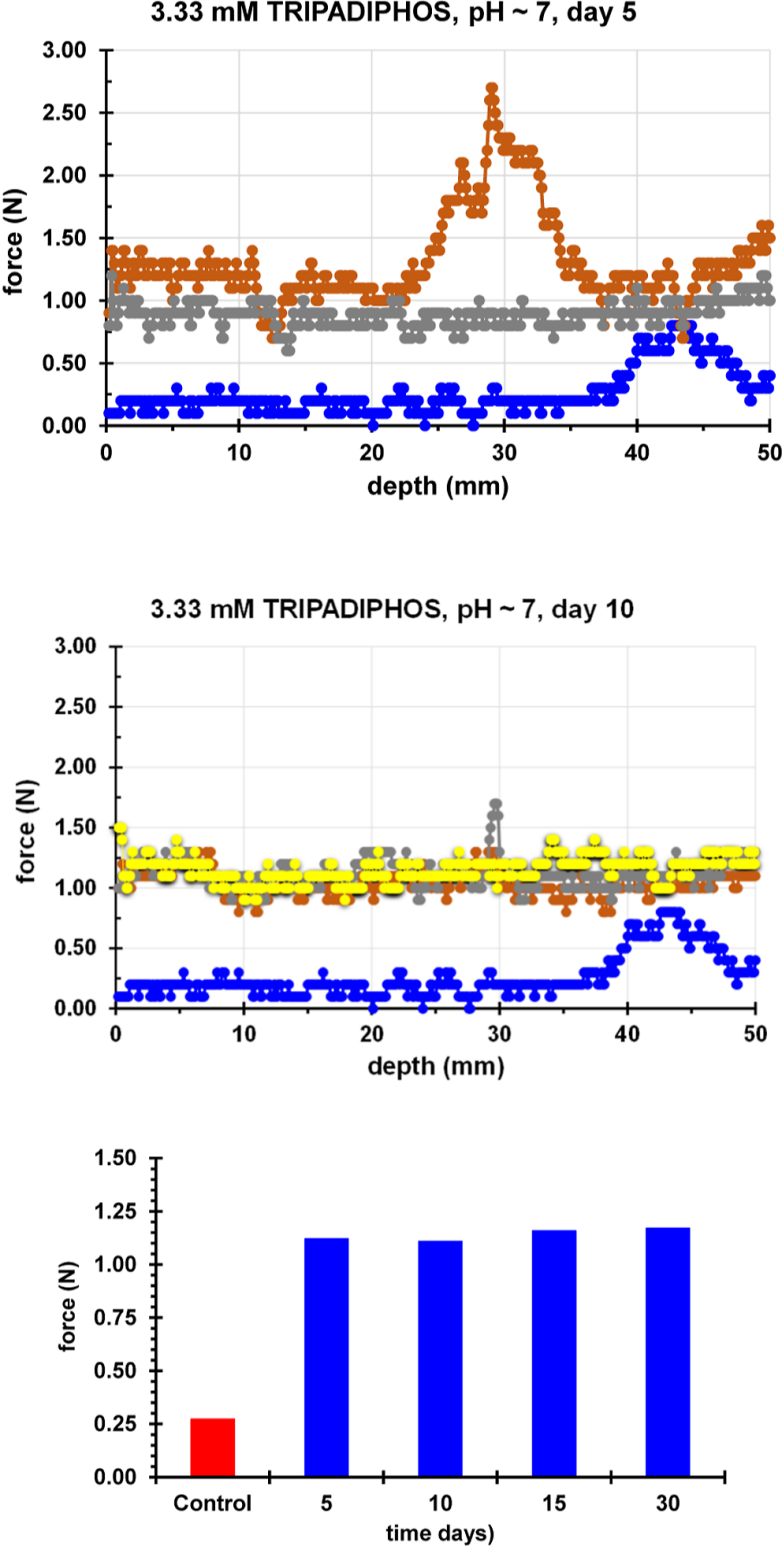
Evaluation of TRIPADIPHOS solution application on artificial gypsum via DRMS. The measurements were taken on the 5^th^ day (upper diagram) and on the 10^th^ day (middle diagram) after consolidant application (0.1 mmol). The lower lines (blue) correspond to the control (untreated) and the upper lines correspond to treated samples (at different drilling spots). The bar graph (lower) shows the insensitivity of the measurements to sample maturation time (average values were used).

Figure 5 presents the DRMS results of the application of TRIPADIPHOS on artificial gypsum specimens. An increase in drilling resistance was noted, from ∼0.25 N (control) to ∼1.05 N (treated), which corresponds to a 320% increase. It is important to note that after the TRIPADIPHOS consolidant is applied the drilling resistance values remain stable after the 5^th^day and up to the 30^th^ day. This is an indication that both Ca-coordination chemistry and polycondensation processes are complete by the 5^th^ day.

## Performance of Alkoxysilane Consolidants

**Figure 6:**
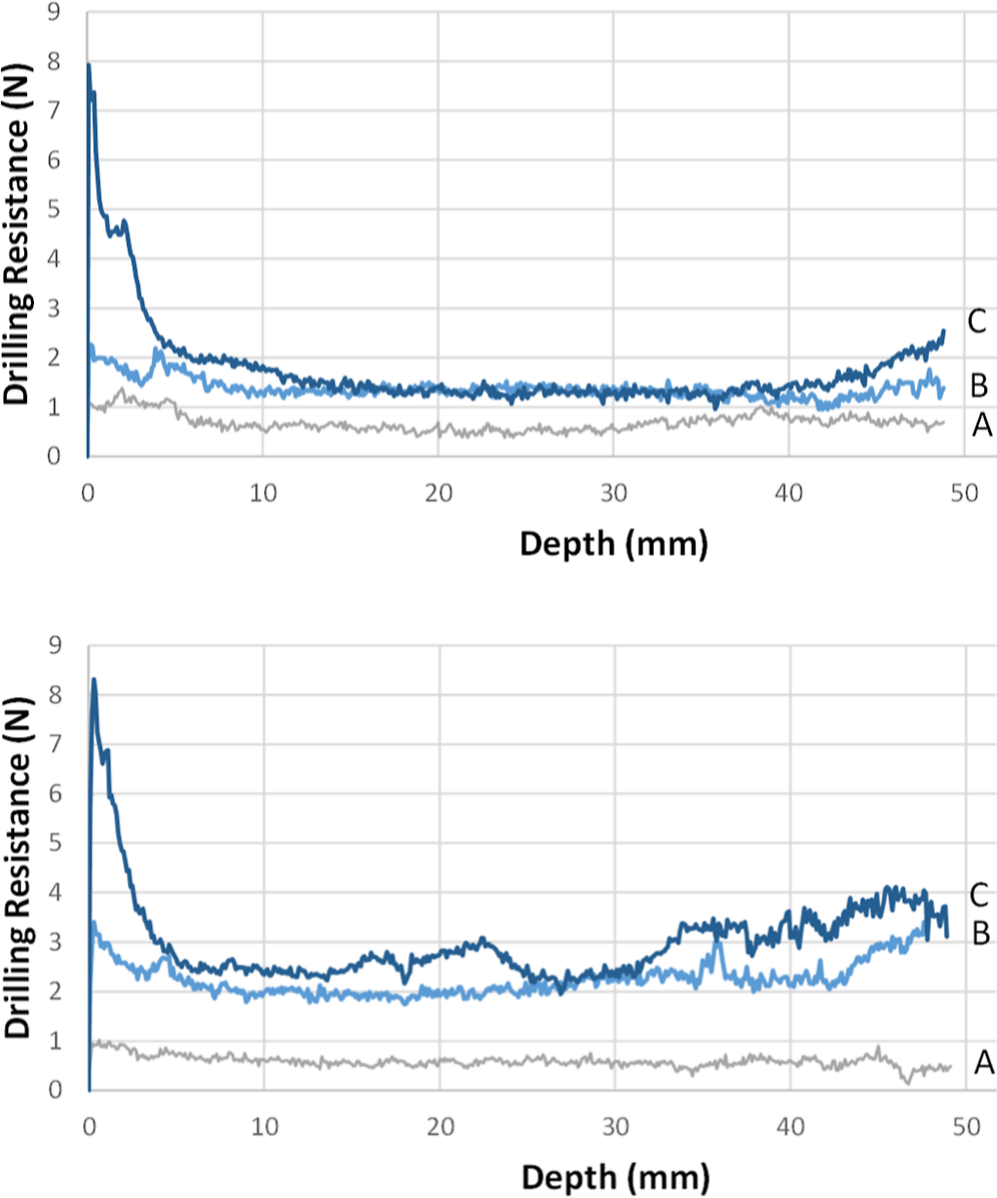
DRMS results acquired from an artificial gypsum sample treated with the consolidants RC-70 (upper) and RC-90 (lower). (A) = untreated sample, (B) = sample after 1^st^ immersion, (C) = sample after 2^nd^ immersion.

In the plots of TEOS-treated samples (Figure 6), the drilling resistance showed an increasing trend following the two successive immersions. In the case of the consolidant RC-70, after the first immersion (B) the average drilling resistance increased to an average value of 1.37 N (98% increase), while after the second immersion (C) the average drilling resistance was found 1.75 N (153% increase), showing a slight additional increase. In the case of the consolidant RC-90, the average drilling resistance after the first immersion (B) increased dramatically, to 2.24 N (280% increase) and to 3.01 N (410% increase) after the second immersion (C), respectively.

It is interesting to note that a high drilling resistance (∼8 N) is observed after the second immersion for both RC-70 and RC-90 consolidants. This is ascribed to the formation of a siliceous “crust” on (or close to) the sample surface, as is corroborated by the SEM study (see below). From a practical standpoint, crust formation is not the optimal consolidation strategy, as it literally covers the surface of the sample, and ultimately offers poor cohesion in the sample interior.

It is worth noting that in both cases after the first immersion, a constant average drilling resistance was reached to a certain extent, throughout the whole profile of the treated samples. After the second immersion, both consolidants demonstrate a significantly increased drilling resistance along the first 5 mm depth. This could possibly be attributed to a decrease in porosity in this regime due to increased condensation of the consolidating compound. This relates to the fact that the sample portion (first 5 mm depth) was in direct contact with the consolidant during the immersion cycles. The data at <5 mm depth were taken into account for the calculation of the average resistance.

### Characterization of Consolidated Gypsum Samples

Consolidated gypsum samples were characterized by selected techniques. The findings are discussed below.

#### Nuclear Magnetic Resonance Spectroscopy

These studies were focused only on artificial gypsum samples consolidated with TRIPADIPHOS. The presence of the consolidant in the interior of gypsum samples was confirmed by ^31^P NMR spectroscopy. The gypsum powder from the DRMS experiments was collected and initially was treated with pure water in order to extract the water-soluble TRIPADIPHOS into the liquid phase, however, these extracts showed no phosphorus signal in the ^31^P NMR spectra. This is an indication that the consolidant is strongly bound to the gypsum surface. Subsequently, more harsh conditions were used to detach it, by means of a concentrated HCl aqueous solution. Again, the aqueous extract was collected and the ^31^P NMR spectrum was recorded, but this time longer acquisition time (∼1 h) was used. A distinct single peak at ∼8.8 ppm appeared (see Figure S7 in the Supporting Information), which is an unequivocal proof that TRIPADIPHOS was indeed attached to the gypsum. The position of the peak virtually matches that of pure TRIPADIPHOS (∼8.4 ppm, see Figure S4 in the Supporting Information). The minor shift is ascribed to the possible pH differences in the two samples, which can affect the precise peak position.

#### Vibrational Spectroscopy (ATR-IR)

The identification of silicatic consolidants in gypsum is problematic because the main characteristic intense band due to Si–O bonds and Si–O–Si bridges strongly overlaps with that of the sulfate ion (spectral range 900–1200 cm^–1^). Further complications may arise due to the fact that the consolidants (either inorganic or organic) are usually in low concentrations, hence their bands in the IR spectrum are commonly of weak intensity. Hence, the IR spectrum of consolidated gypsum with RC-70 (pure TEOS) shows no differences with that of the control (pure gypsum). The presence of RC-90 (TEOS + methylphenylsilicone resin) can be identified by a number of weak bands in the region 750–1000 cm^–1^ (that are absent in the pure gypsum spectrum) and are assigned to the Si–CH_3_ and Si–Ph moieties of the resin,^33^ see Figure S8 in the Supporting Information. Very weak (but clearly identifiable) bands in the 2900–3100 cm^–1^ region are due to the C–H stretching vibrations of the resin (Figure S8 in the Supporting Information). Unfortunately, no discernible bands due to TRIMEPHONA or TRIPADIPHOS were identified in the IR spectra of treated gypsum samples (see Figure S9 in the Supporting Information).

#### X-ray Powder Diffraction

All tested consolidants generate gels or solids within the gypsum pores, so no peaks assigned to them should appear in the XRD patterns of consolidated gypsum samples. This was confirmed experimentally, see Figure S10 in the Supporting Information. No phase transitions of gypsum were identified.

#### Electron Dispersive Spectrometry

Powdered samples of consolidated gypsum were studied by EDS. Pure gypsum was also studied as a reference and the elements Ca and S were identified in their expected concentrations with a Ca/S atom ration of 1:1 (see Figure S11 in the Supporting Information). In the case of RC-70 and RC-90 consolidants the presence of Si was identified (besides the expected Ca and S, see Figure S12 in the Supporting Information). In the case of the consolidant TRIMEPHONA, in addition to Si, P was also identified at a Si/P atom ratio of ∼1:1, as expected, based on the structure of the molecule (see Figure S13 in the Supporting Information). Finally, the presence of TRIPADIPHOS in the consolidated gypsum specimens was proven by the presence of Si and P in a Si/P ∼ 1:2 atom ratio, as expected, based on the structure of the molecule (see Figure S14 in the Supporting Information).

#### Scanning Electron Microscopy

**Figure 7:**
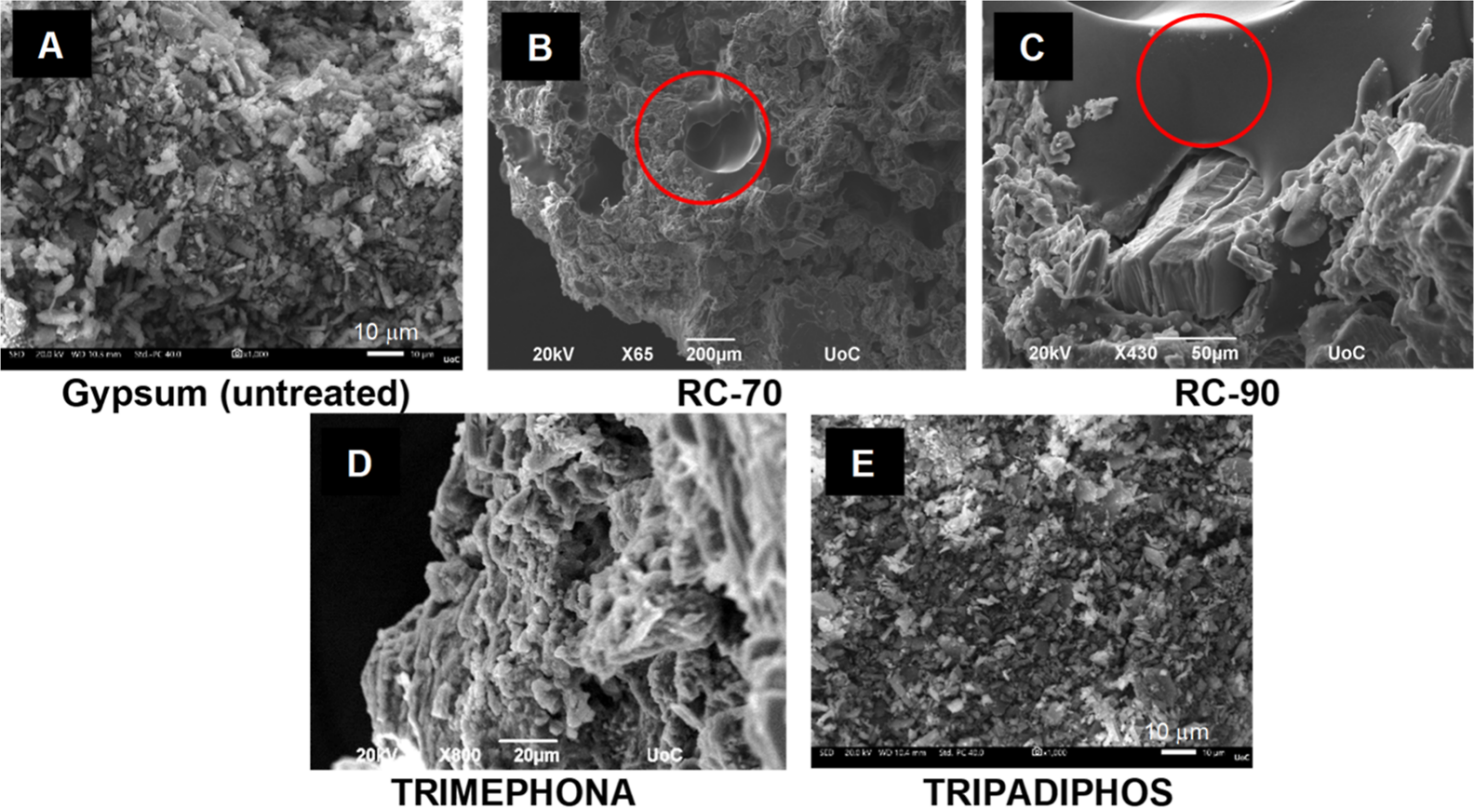
SEM images of untreated gypsum and treated with different consolidants, as shown. The red circles indicate the localized action of RC-70 and RC-90. Note the different scale bars.

Consolidated gypsum samples were also studied by SEM (see Figure 7). In the absence of consolidant, distinct particles of ∼10 μm size are observed (image A). In the presence of RC-70 or RC-90 consolidant-rich gel-like areas are observed (images B and C, respectively; note the red circles), indicating a localized action. It seems that these cosolidants act mainly as fillers. The SEM results corroborate those of the DRMS measurements. The presence of TRIMEPHONA offers some cohesion (image D) but leaves some gaps in the solid surface. The effect of TRIPADIPHOS (image E) creates a smoother, continuous gypsum surface.

**Figure 8:**
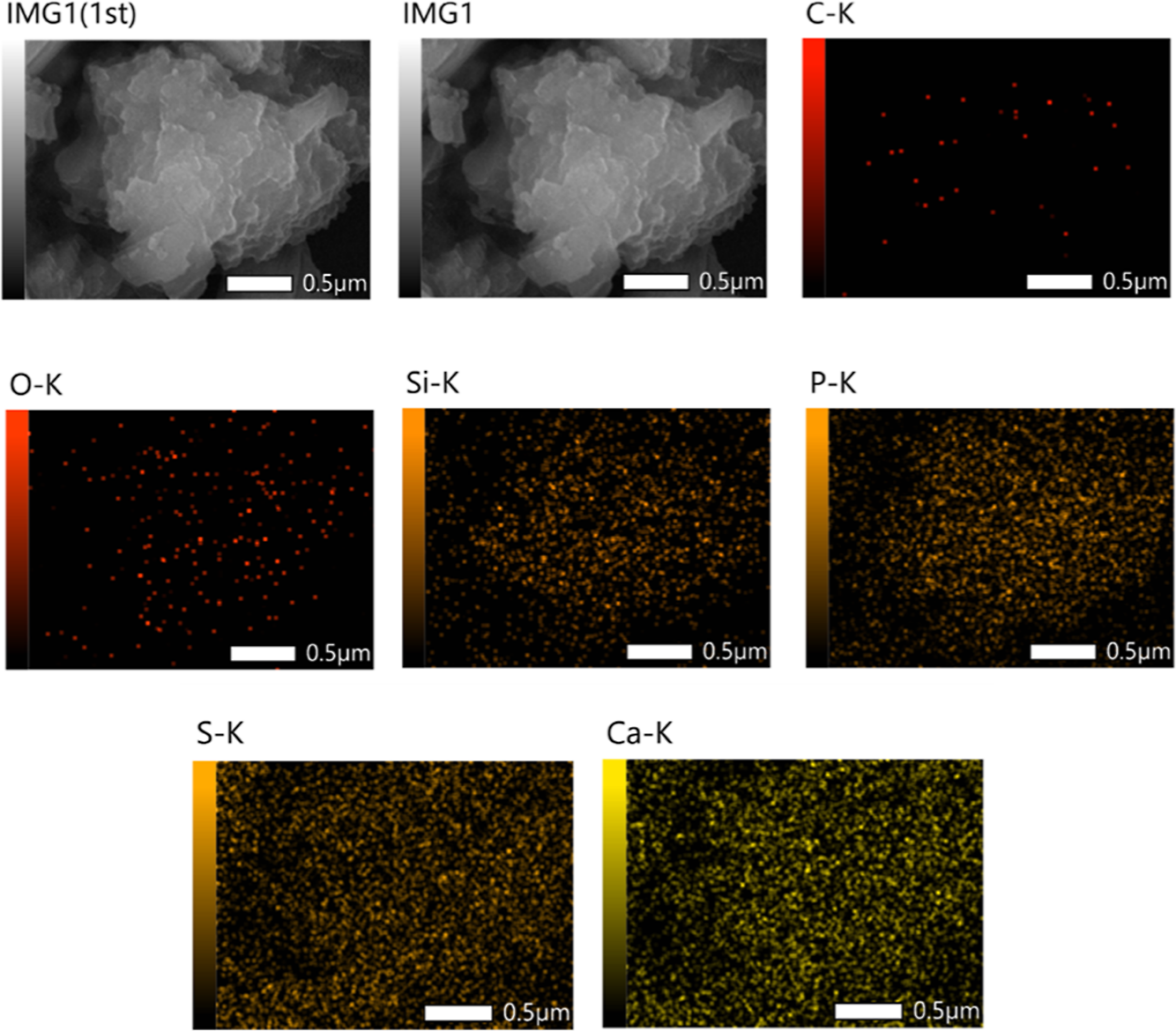
Elemental mapping of the surface of TRIPADIPHOS-consolidated gypsum sample confirming the presence of both Si and P.

The case of TRIPADIPHOS was studied in more detail. We performed elemental mapping on several gypsum particles. The results for one (size ∼2 μm) are presented in Figure 8, as a representative example. Besides the presence of the expected elements Ca, S and O due to gypsum, it is evident that C, Si, and P are also present, due to the TRIPADIPHOS consolidant. It is important to note that Si and P are spread out in the sample. This is a strong indication that TRIPADIPHOS is evenly distributed throughout the gypsum pores and does not have a localized action (like the one observed for RC-70 and RC-90). An additional feature that is evidenced by the elemental mapping results is that TRIPADIPHOS penetrates the gypsum pores and its −Si(OH)_3_ moiety polycondenses in a homogeneous manner. This is important for practical consolidation applications where the consolidant solution is applied on the stone surface (commonly by a brush). It is desired that the consolidant does not get immobilized on the surface (or very close to it), but to penetrate as deeply as possible into the stone interior.

### Crystal Structures of the Model Compounds Ca–C2D and Ca-3D

The binding of TRIPADIPHOS on surface Ca^2+^ locations is a challenge to assess at the atomic level. Hence, in order to investigate the possible binding modes of the amino-di(methylenephosphonate) unit to Ca^2+^ the crystal structures of Ca–C2D and Ca-3D were determined. Both diphosphonates were selected because of their structural similarities to TRIPADIPHOS as far as the amino-di(methylenephosphonate) portion of the molecule is concerned, and their comparable molecular size.

#### Crystal Structure of Ca–C2D

**Figure 9:**
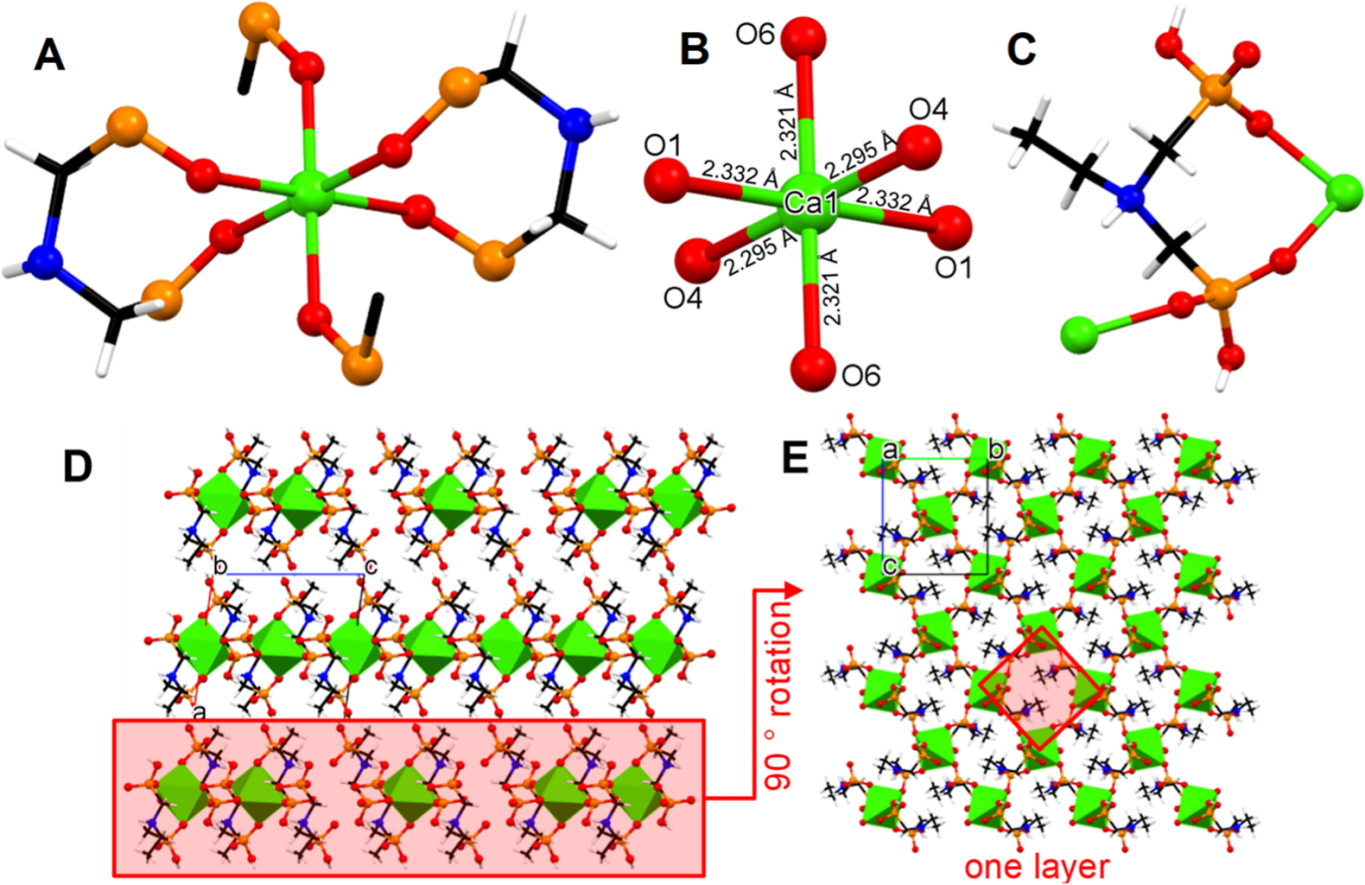
Various views of the structure of Ca–C2D: (A) coordination environment of Ca^2+^. (B) Ca–O bond distances. (C) Binding of the C2D ligand. (D) The layered structure of Ca–C2D viewed along the *b* axis. (E) Partial view of one layer viewed along the *a* axis. Color codes: Ca green, P orange, O red, N blue, C black, H white.

The crystallographically unique Ca^2+^ center is located in a slightly distorted octahedral environment (confirmed by the SHAPE software),^34^ with O–Ca–O angles ranging from 80.95 to 94.33°, see Figure 9A. The Ca^2+^ is coordinated by six O phosphonate atoms. There are two identical 8-membered chelating rings that form the basal plane of the octahedron. Each is formed by one C2D ligand offering one P–O^–^ moiety from different phosphonate groups. In spite of the inherent instability of an 8-membered chelating ring, this structural motif is not uncommon in metal phosphonate chemistry.^35^ The coordination sphere of the Ca^2+^ is completed by two P–O^–^ moieties in a trans position, coming from two different C2D ligands. The Ca–O bond distances are found in the range 2.295–2.332 Å (Figure 9B), which are expected and noted for other Ca–phosphonate compounds.^35^ The Ca/C2D molar ratio in the structure is 1:2, with each C2D ligand binding two Ca^2+^ cations (Figure 9C), and one phosphonate acting as a bridge and the second being terminal.^36^ Each phosphonate group is singly deprotonated (due to the low pH of synthesis), and the N is protonated due to its high basicity. This renders the C2D ligand with an overall charge of “–1”. Two C2D ligands are used to achieve electroneutrality with one Ca^2+^ center. The structure of Ca–C2D is 2D layered with interlayer Ca···Ca distances at 10.067 Å (Figure 9C). The 2D polymeric structure is propagated within the layer by the chelating/bridging phosphonate group. The second, terminal phosphonate moiety extends in the interlayer region, along with the –N(H)CH_2_CH_3_ aliphatic chain. Each set of four closest Ca centers form a rhomb (Figure 9D). These consecutive rhombs along the *a* axis form a channel, however it is “filled” with the –N(H)CH_2_CH_3_ aliphatic chains, hence, the structure is not porous. There are no water molecules in the structure.

#### Crystal Structure of Ca–C3D

**Figure 10:**
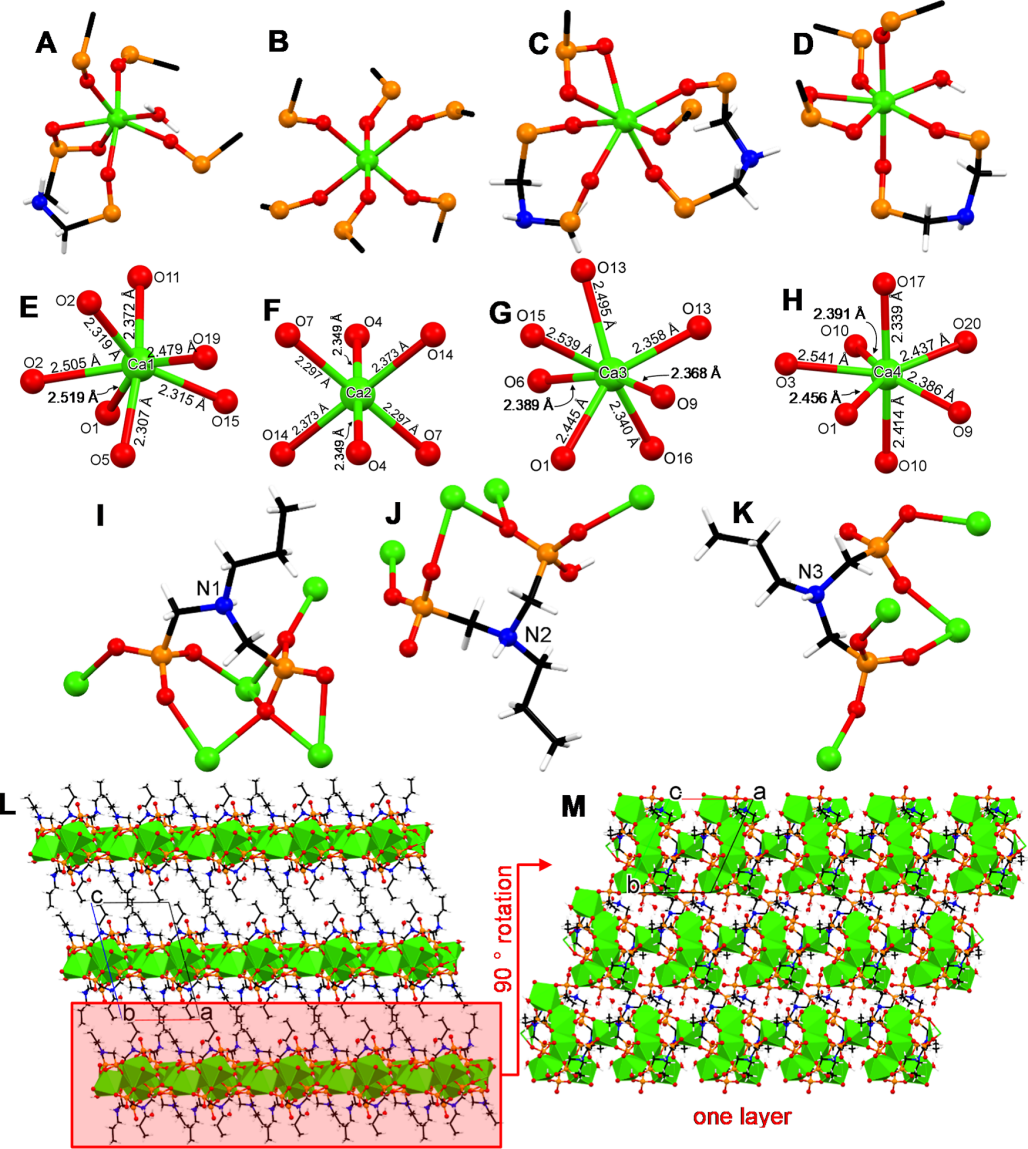
Various views of the structure of Ca–C3D: (A–D) coordination environment of the four different Ca^2+^ centers. (E–H) Ca–O bond distances. (I–K) binding modes of the three different C3D ligands. (C) Binding of the C2D ligand. (L) The layered structure of Ca–C3D viewed along the *b* axis. (M) Partial view of one layer viewed along the *c* axis. Color codes: Ca green, P orange, O red, N blue, C black, H white.

The ligand C3D possesses only one additional –CH_2_– group compared to C2D. Nevertheless, the structure of Ca–C3D is profoundly different from that of Ca–C2D. There are four distinct Ca centers (Figure 10A). Their coordination geometries (according to the SHAPE software) are Ca1 and Ca4 pentagonal bipyramid, Ca2 octahedron, Ca3 capped trigonal prism. Each Ca center presents its own structural and coordination idiosyncrasies, so they will be examined separately.

The coordination geometry of 7-coordinated Ca1 is pentagonal bipyramid. It is bound by three terminal phosphonate oxygens [O2(P1), O11(P4), O15(P5)] in a meridional arrangement. In addition, a 4-membered chelating ring is formed by O1 and O2 of the same phosphonate group (P1), while O5 (of the other phosphonate group P2, but in the same C3D ligand) coordinates to Ca1. Hence, this C3D ligand (identified by the N1 nitrogen) acts in a tridentate fashion for Ca1. The coordination sphere of Ca1 is completed by a water molecule (O19).

The coordination geometry of 6-coordinated Ca2 is octahedral. It is bound by six terminal phosphonate oxygens [2 × O4(P2), 2 × O7(P3), and 2 × O14(P5)], belonging to three different C3D ligands. Neither chelating rings nor water molecules are present around Ca2.

The coordination geometry of 7-coordinated Ca3 is capped trigonal prism. Besides the terminally bound O9 (from P3) all other types of coordination are chelating. Specifically, O13 and O15 (both from the same P5) create a 4-membered chelating ring with Ca3. There are two nearly identical 8-membered chelating rings formed by two different (N1 and N3) C3D ligands. Each one is generated by two oxygens from different phosphonate groups (but within the same C3D ligand).

The coordination geometry of Ca4 is pentagonal bipyramid, just like Ca1, but with differences in the individual binding of the donor atoms. Specifically, phosphonate oxygens O10 (from P4) and O17 (from P6) are terminal. O1 and O3 (both from P1) form the familiar 4-membered ring with Ca4. There is one 8-membered chelating ring formed by O9 (from P3) and O10 (from P4), both from the same (N2) C3D ligand. The coordination sphere of Ca4 is completed by a water molecule (O20).

There are three distinct types of C3D ligands (identified by their N atoms, N1, N2, and N3). Their binding modes are shown in Figure 10I,J,K. In all three the N atom is protonated. In ligand N1 both phosphonate groups (P1 and P2) are doubly deprotonated, each contributing a “–2” charge, hence the overall charge of this ligand is “–3”. All three oxygens of P2 (O4, O5, O6) are bound to three Ca centers in a terminal fashion. All three oxygens of P1 (O1, O2, O3) are bound to four Ca centers in a variety of binding modes. O1 is triply bridging three Ca centers. O2 connects two Ca centers, while forming a 4-membered chelating ring with O1. O3 bridges two Ca centers, while forming a 4-membered chelating ring with O1. Ligand N1 overall binds five Ca centers.

In ligand N2 the phosphonate group P4 is doubly deprotonated, contributing a “–2” charge, while the phosphonate group P3 is singly deprotonated, with a “–1” charge. Hence, taking into account the protonated N atom, the overall charge of this ligand is “–2”. The oxygens O10 and O11 (of P4) are each terminally bound to a Ca center, leaving the O12 noncoordinating (it is the P=O phosphoryl moiety). The O9 (of P3) is bridging two Ca centers, while O7 (of P3) is terminally coordinating one Ca center. The O8 (of P3, it is the P–O–H moiety) is noncoordinating. The O9 (of P3) and O10 (of P4) form an 8-membered chelating ring. Ligand N2 overall binds four Ca centers.

Lastly, in ligand N3 both phosphonate groups P5 and P6 are doubly deprotonated, each contributing a “–2” charge. Hence, taking into account the protonated N atom, the overall charge of this ligand is “–3”. All three oxygens of P5 (O13, O14, O15) are bound to three Ca centers in a terminal fashion. The oxygens O16 and O17 (of P6) are each terminally bound to a Ca center, leaving the O18 noncoordinating (it is the P=O phosphoryl moiety). The O13 (of P5) and O16 (of P6) form an 8-membered chelating ring. Ligand N3 overall binds four Ca centers.

The structure of Ca–C3D is a neutral coordination network. Electroneutrality is achieved by balancing the positive and negative charges in the following way. The overall positive charge includes the four distinct Ca centers [4 × (+2) = +8 charge] and the three protonated N atoms [3 × (+1) = +3 charge], yielding a “+11” cationic charge. This is counterbalanced by the overall “–11” negative charge on the N1 (“–4” charge), N2 (“–3” charge) and N3 (“–4” charge) ligands.

### Performance Comparisons between Consolidants

In the previous sections the performance of all four consolidants was presented. However, caution must be exercised in interpreting these results because erroneous conclusions may be drawn. The different absolute drilling resistance values need to be examined by taking into account the actual concentration of the consolidant in the fluid used to penetrate the artificial gypsum samples. The consolidants RC-70 (30% TEOS), RC-90 (24% TEOS + 6% resin) and TRIMEPHONA (50% active substance) are used “straight out of the bottle” without any dilutions. The consolidant TRIPADIPHOS exists in a solid form, so preparation of appropriate stock solutions was necessary. Two concentrations were prepared: 3.33 mM (0.1 mmol of the compound in 30 mL of water) and ∼10 mM (0.3 mmol of the compound in 30 mL of water). The results with the higher concentration did not cause an enhancement of drilling resistance, compared to the lower concentration.

**Figure 11:**
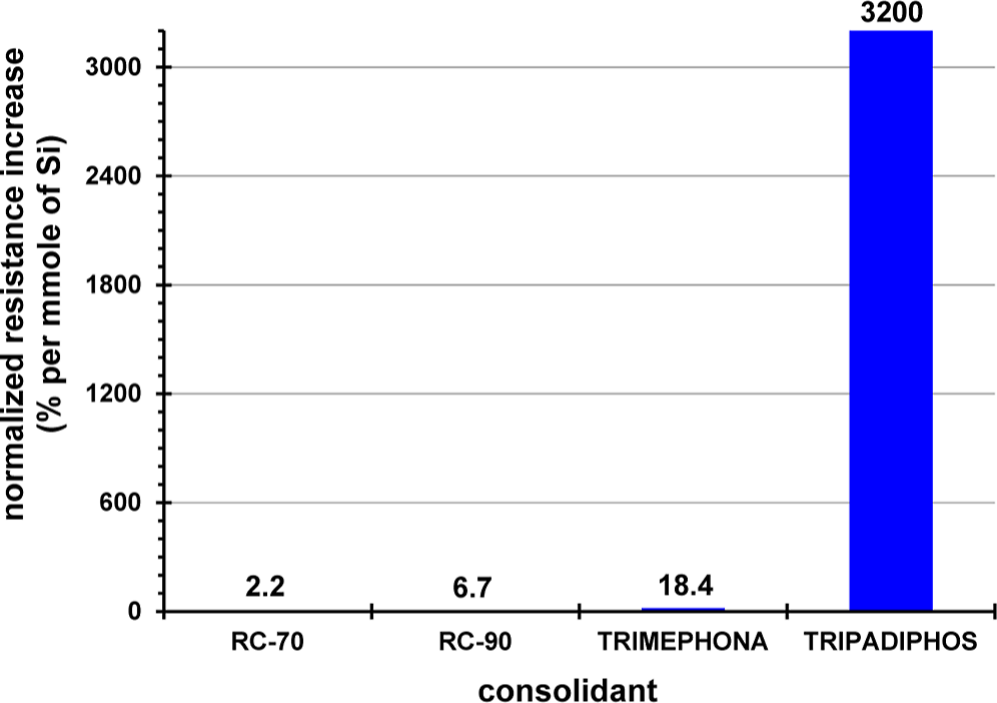
Comparison of the efficacy of the four tested consolidants based on the DRMS measurements on artificial gypsum samples. This comparison is based on the % increase in drilling resistance per mmol of Si.

In order to make a reliable comparison, the drilling resistance values must be normalized in some way. We chose to do that by expressing the “% drilling resistance increase” per “mmol of Si in the pure consolidant”. In this way, it is possible to make reliable performance comparisons at the molecule level. Figure 11 presents a comparison bar graph between the four consolidants and it is useful for performance ranking purposes. The reader is cautioned that the values plotted are not actual measurements, but are based on experimental data, appropriately normalized. It is evident that the consolidant TRIPADIPHOS outperforms the other three consolidants in a dramatic way.

In an effort to interpret the obtained results and the differences in drilling resistance enhancement, we need to look at the chemical structures of the consolidants. RC-70 (containing only TEOS) shows low drilling resistance enhancement. Once TEOS penetrates the pores of the gypsum material it undergoes hydrolysis and concomitant polycondensation reactions. The hydrolysis reaction produces silicic acid [Si(OH)_4_] and four equivalents of ethanol as byproduct. The silicic acid then undergoes complex polycondensation reactions, in which Si–O–Si bonds are formed and the amorphous silica network is built. The low drilling resistance enhancement in the case of RC-70 is not unexpected, and the silica xerogel that forms eventually simply acts as a “filler” of the gypsum pores. Similar events occur in the case of RC-90, with the difference that now an unreactive resin is present, which simply enhances to a minor extent the “filler” effect of hydrolyzed/condensed TEOS.

The multifunctional consolidant TRIMEPHONA demonstrates *a* ∼ 9× performance increase compared to RC-70 and *a* ∼ 3× performance increase compared to RC-90 (see Figure 11). We assign this improvement to the effect of the phosphonate group that can attach to exposed Ca^2+^ centers on the pore surface. However, the ability of the phosphonate group to interact with the Ca^2+^ centers is hampered by the fact that there is only one such group available per consolidant molecule and that the presence of a −CH_3_ substituent on the P atom (see Figure 2) may stereochemically hinder this interaction.

The multifunctional consolidant TRIPADIPHOS shows an extremely high increase in performance compared to all other consolidants. We assign this superior performance to the following attributes:

(a) TRIPADIPHOS contains two phosphonate groups that are highly polar, anionic (at the pH of application) and possess high affinity for surface Ca^2+^ sites of gypsum. This phenomenon is well documented in the literature.^37^
(b) The positioning of the phosphonate groups allows the molecule to bind to either one surface Ca^2+^ site (in a bidentate fashion) or two adjacent surface Ca^2+^ sites (in a bridging fashion). Such interactions have been invoked before.^38^ These binding motifs enhance the strength of the Ca–phosphonate interaction and anchor the consolidant more strongly onto the gypsum surface.
(c) The silane triol [−Si(OH)_3_] portion of the molecule can undergo condensation reactions with neighboring silane triol moieties, which lead to building a Si–O–Si network that fills the pores and stabilizes the gypsum stone.^39^

**Figure 12:**
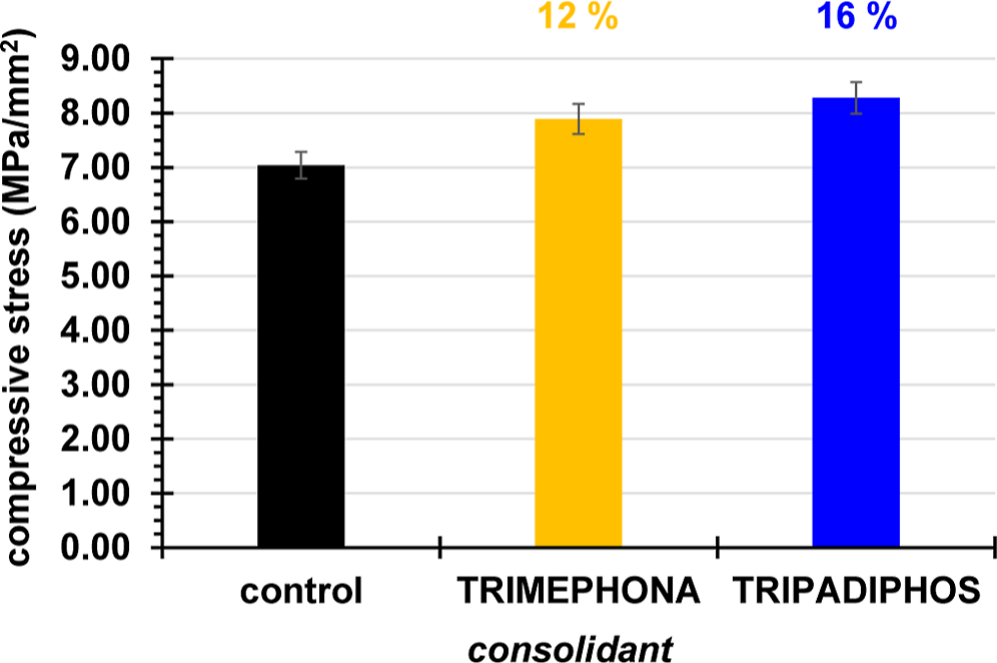
Compressive stress results from “control” gypsum specimens, and gypsum treated with TRIMEPHONA and TRIPADIPHOS.

To further evaluate the consolidation action of the consolidants, compressive strength measurements were performed on gypsum samples treated with TRIMEPHONA and TRIPADIPHOS and compared to “control” gypsum (no consolidants present). Four measurements were performed per group. Compressive stress values were calculated. These results are shown in Figure 12. It is noted that both consolidants show a small, but measurable increase in compressive stress values (+12% for TRIMEPHONA and +16% for TRIPADIPHOS). It is not as impressive as the increase observed in the DRMS data, which means that the consolidants only marginally improve the resistance of gypsum to compression, as well as its elasticity.

**Figure 13:**
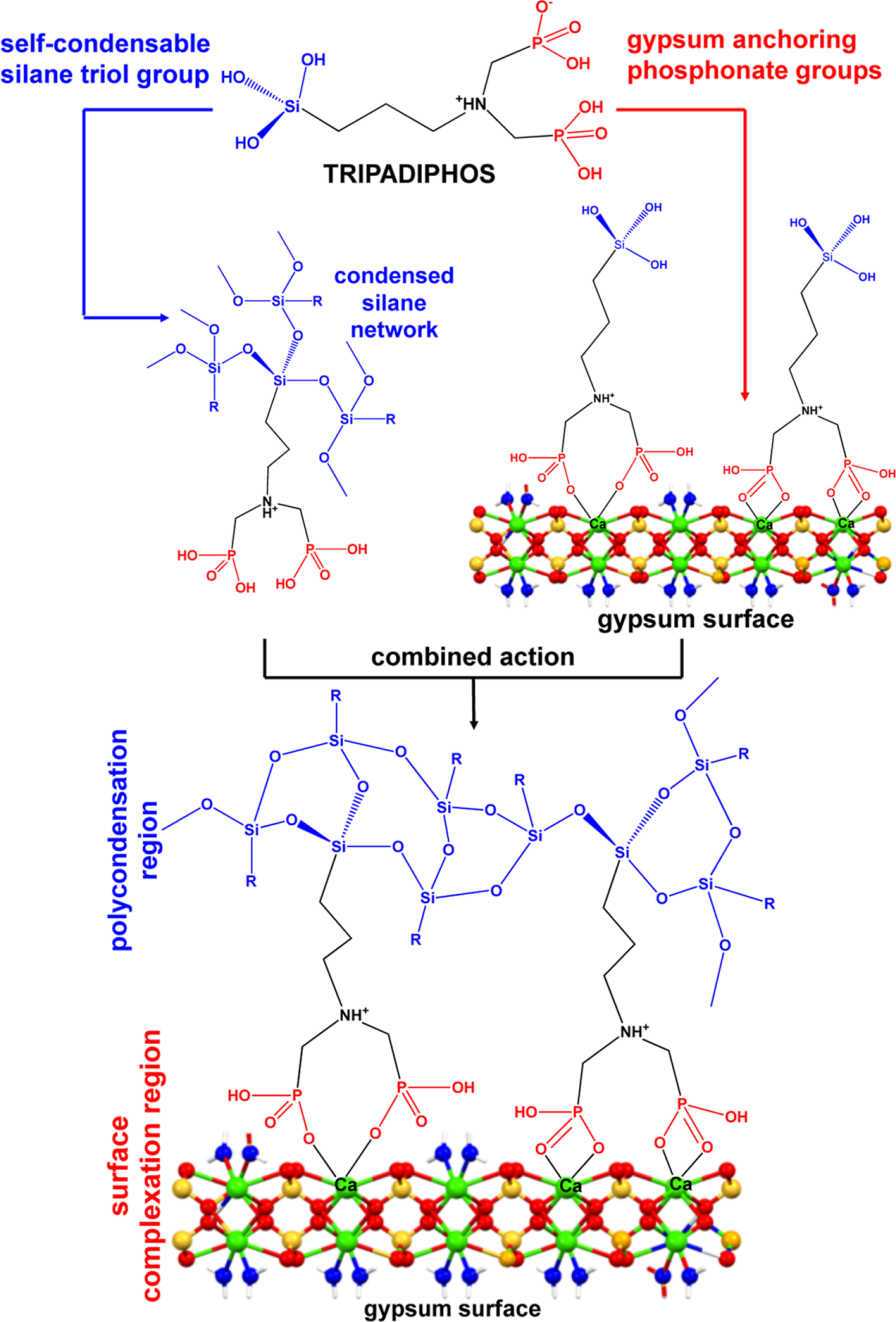
Conceptual correlation of the structural/functional features of TRIPADIPHOS with its efficacy as a gypsum consolidant. Color codes: calcium, green; oxygen, red (the Ca-bound waters are differentiated in blue in the gypsum layer image); hydrogen white; sulfur, yellow.

Figure 13 summarizes visually the above arguments and shows how the multifunctional features of TRIPADIPHOS are operational in gypsum stone consolidation.

### Structural and Geometrical Correlations between the Ca Sites in the Structures of Gypsum and the Ca–Phosphonates Ca–C2D/Ca-3D

The Ca site in gypsum resides in a triangular dodecahedral (TDD-8, *D*_2d_) environment, according to SHAPE. Two of the sulfate anions (situated trans to each other) are bidentate and form 4-membered rings with Ca. Two sulfate ions are monodentate and are cis to each other. The coordination sphere of Ca is completed by two water molecules that are in cis positions and extend toward the interlayer space. Upon mild thermal treatment (the process starts at ∼60 °C)^40^ a facile transformation of gypsum to hemihydrate (CaSO_4_·1/2H_2_O) takes place. This is an indication that the Ca-bound water molecules can rather easily be removed from gypsum.

As indicated above, the working hypothesis of this work is that a Ca-binding anionically charged consolidant can replace the surface Ca-bound waters in gypsum, while its negatively charged moieties coordinate to the exposed Ca^2+^ centers. A high-performance consolidant does not have to substitute the water molecules in every exposed Ca^2+^ cation, and it seems that partial replacement is sufficient. The role of the Ca-binding moieties is played by the phosphonate groups in TRIPADIPHOS. The precise mode of binding of its phosphonate groups to the Ca^2+^ centers is impossible to discern. However, one can investigate certain geometrical features of the Ca site in gypsum and draw correlations with similar Ca coordination environments in crystallographically characterized Ca–phosphonate networks, such as the “model” compounds Ca–C2D and Ca–C3D. We have selected the H_2_O–Ca–OH_2_ angle in gypsum (98.40°) as the geometrical criterion for comparisons to analogous O–Ca–O angles of the various Ca sites in the structures of Ca–C2D and Ca–C3D. Only the acute O–Ca–O angles (<100°) have been considered. The values of these angles can reveal (even qualitatively) whether phosphonate binding to Ca in gypsum is geometrically possible.

**Figure 14:**
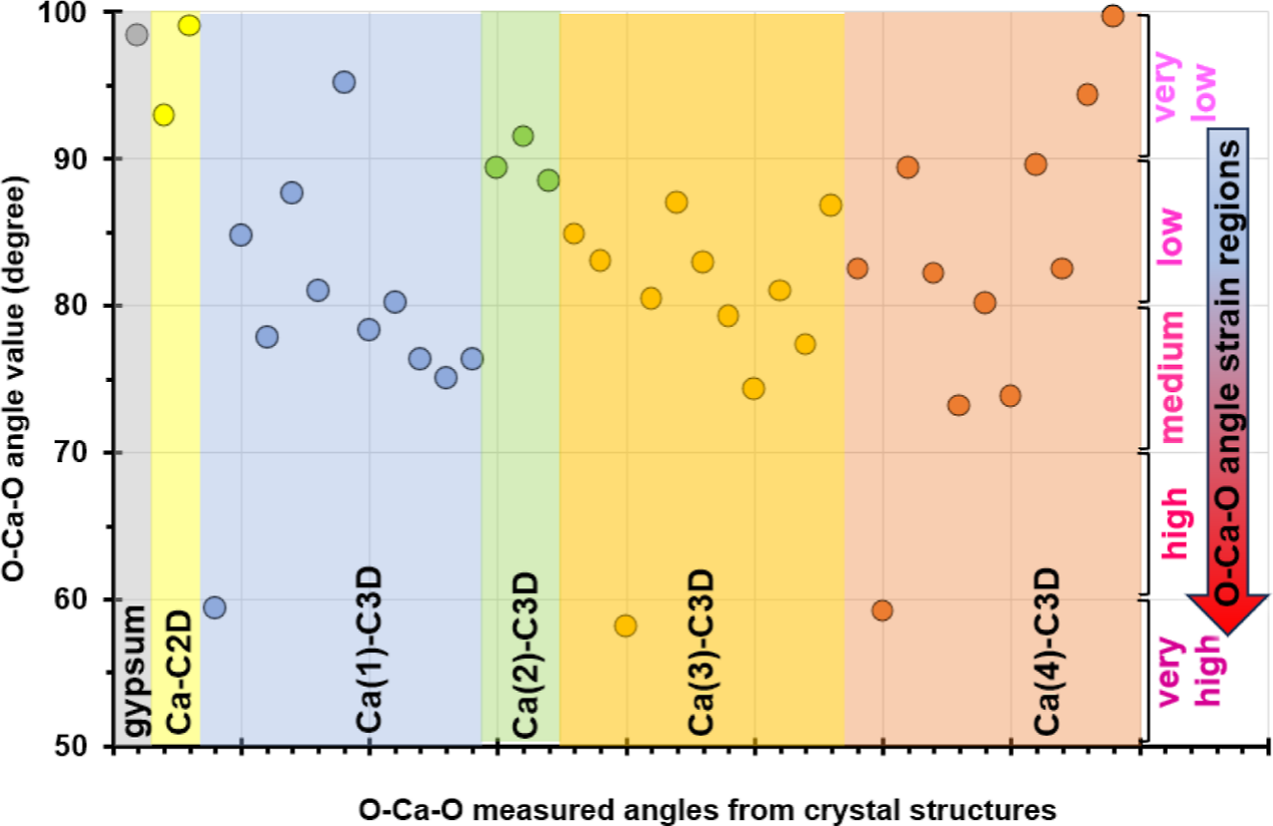
Mapping of all acute O–Ca–O angles (<100°) in the crystal structures of gypsum (gray), Ca–C2D (yellow) and Ca–C3D (Ca1 light blue, Ca2 light green, Ca3 light orange, and Ca4 dark orange). The O–Ca–O angles have been categorized based on their deviation from the O–Ca–O angle in gypsum as “very low” (90–100°), “low” (80–90°), “medium” (70–80°), “high” (60–70°) and “very high” (50–60°) strain (see the right portion of the graph).

Hence, Figure 14 represents a mapping of all such angles recorded in the structures of Ca–C2D and Ca–C3D. The values have been color-coded and placed in such a way as to reflect their Ca center. Ca-2D has one type of Ca, whereas Ca-3D has four types of Ca centers (see also Figure 10). The angles have also been categorized based on their deviation from the O–Ca–O angle in gypsum as “very low” (90–100°), “low” (80–90°), “medium” (70–80°), “high” (60–70°) and “very high” (50–60°) strain (see the right portion of the graph).

**Figure 15:**
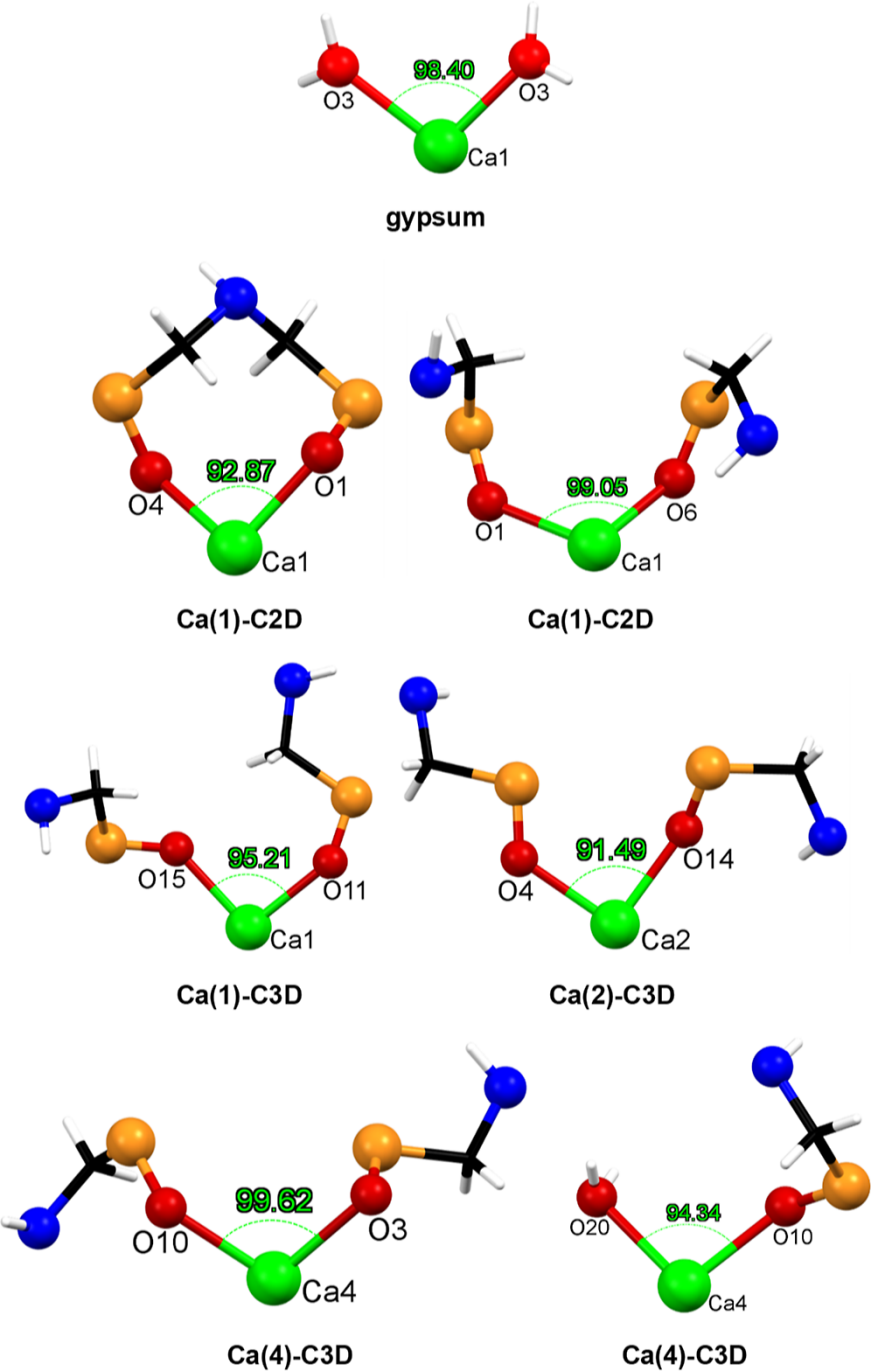
Profiling of all Ca-binding modes with the O–Ca–O angle values in the “very low” strain region (see also Figure 14).

The measured O–Ca–O angles range from 58.12° to 99.62°. The smallest values originate from a bidentate phosphonate coordination with the formation of a Ca–O–P–O 4-membered ring (very high strain region in Figure 14). This feature is present in the coordination sphere of Ca1, Ca2, and Ca4 of Ca–C3D. The highest values range from 91.49 to 99.62° (low strain region in Figure 14) and these are very close to the analogous angle in gypsum. This feature is present in the coordination sphere of Ca1, Ca2, and Ca4 of Ca–C3D. It is reasonable to assume that the larger the deviation of a specific O–Ca–O angle in the two Ca–phosphonates from that of gypsum, the higher the angle strain. Hence, we propose that the O–Ca–O angles in the low strain region reveal the most favorable Ca–phosphonate binding modes to the Ca sites on gypsum. Figure 15 depicts all these possible coordination modes together with the angle values. The O1–Ca1–O4 angle (92.87°) is the result of a bidentate chelating mode of both phosphonates of one C3D ligand on a single Ca, forming an 8-membered ring. There are four monodentate binding motifs in which two phosphonate moieties from two different C3D ligands coordinate to the same Ca (O1–Ca1–O5 99.05°, O11–Ca1–O15 95.21°, O4–Ca2–O14 91.49°, and O3–Ca4–O10 99.62°). Lastly, there is a single monodentate binding of only one phosphonate group to Ca, leaving one water molecule in place (O10–Ca4–O20 94.34°). Several O–Ca–O angle values fall in the intermediate strain regions (low, medium, and high, in the range 73.19–89.51°). Certainly, these may reflect possible binding motifs of the phosphonate groups to Ca, but their increasing angle strain may render them less likely to occur. All measured O–Ca–O angles are given in Table S1 in the Supporting Information.

Based on the above considerations we propose that the preferred binding mode of TRIPADIPHOS onto surface Ca centers on gypsum is the 8-membered chelating ring, demonstrated by an O1–Ca1–O4 angle of 92.87°, very close to that of gypsum. An additional argument that corroborates this proposal is that only one diphosphonate molecule is required for grafting, whereas all other monodentate interactions require two condolidant molecules. Finally, the monodentate binding of a single phophonate group, in the presence of a water molecule on Ca suggests that TRIPADIPHOS may also exhibit this type of grafting. In the absence of precise structural information at the molecular level, it is impossible to pinpoint which binding mode takes place during the consolidation process. It would be safe to assume that several binding motifs occur at the same time during the consolidation of gypsum by TRIPADIPHOS.

Based on the results presented above and the structural considerations and correlations, we propose a possible scenario for the consolidating action of TRIMEPHONA on gypsum. This is outlined below in several successive steps.

**Figure 16:**
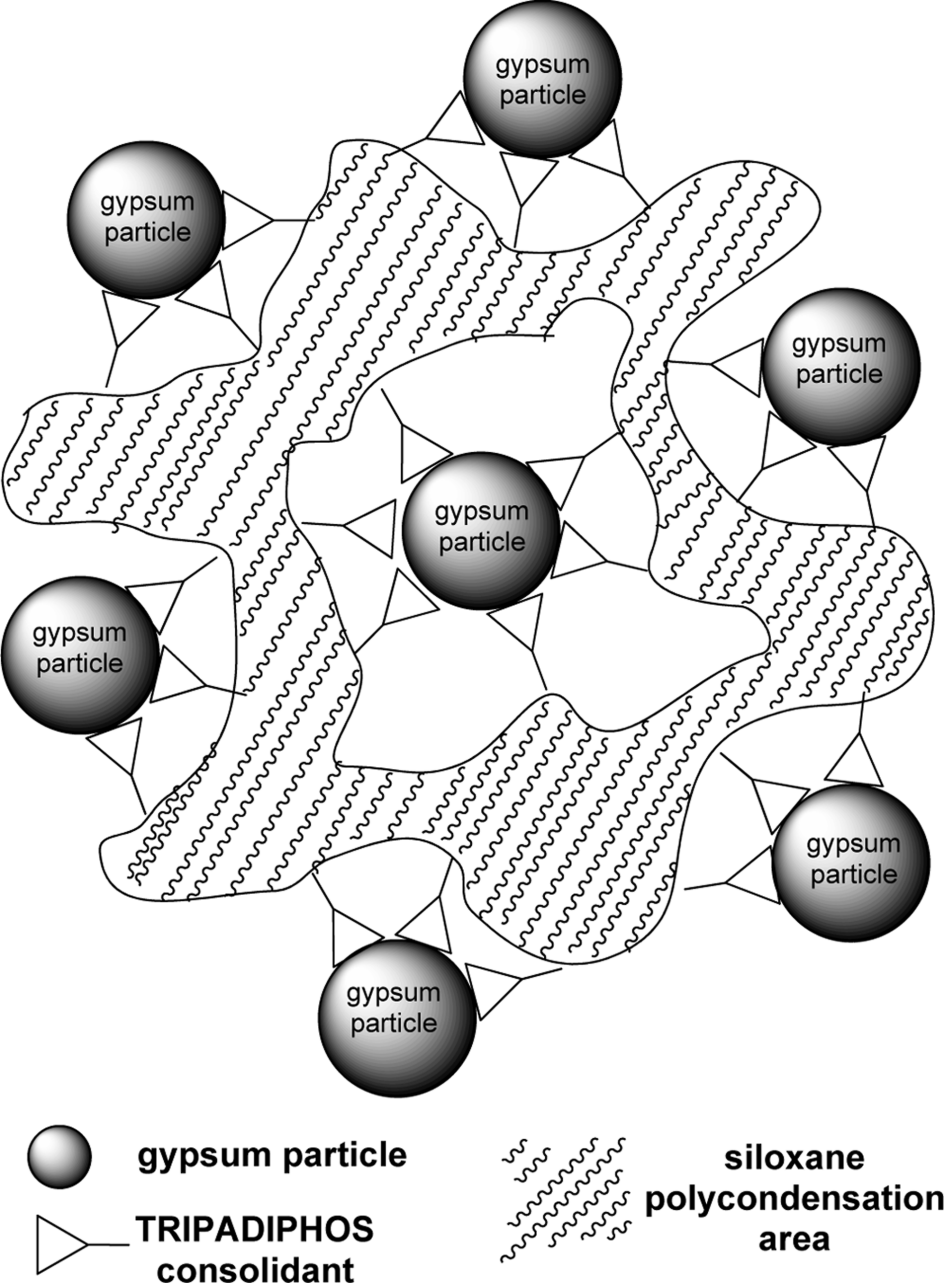
Schematic representation of the consolidating action of TRIPADIPHOS on gypsum.

(a) The consolidant TRIMEPHONA enters the interior of the gypsum sample due to capillary action.
(b) The consolidant anchors itself to the Ca sites of the gypsum surface via surface complexation. Possible modes of phosphonate binding include the formation of an 8-membered chelating ring (bidentate coordination) by replacing the two water molecules, monodentate coordination by replacing only one water, and doubly monodentate coordination by replacing both waters. However, a combination of the above-mentioned binding modes cannot be excluded.
(c) The −Si(OCH_2_CH_3_)_3_ groups begin to get hydrolyzed to form the silanetriol −Si(OH)_3_ moiety. This step may occur simultaneously as step (b).
(d) The silanetriol −Si(OH)_3_ moieties generated by hydrolysis during step (c) start to polycondense, forming Si–O–Si bridging units that connect the TRIPADIPHOS molecules forming a continuous network of siloxane bonds.
(e) An extended siloxane network forms, which not only fills the microvoids within the gypsum surface, but also is connected chemically to the gypsum surface via Ca–phosphonate coordination bonds, acting as a functional “glue” that binds the gypsum particles together. Figure 16 provides a visual representation of this network.

## Conclusions

In the present paper, the efficiency of four consolidants for gypsum specimens was presented and evaluated based on drilling resistance measurements (DRMS). Two of them (RC-70 and RC-90) are alkoxysilane-based and they are considered as benchmark consolidants. The other two [3-(trihydroxysilyl)propyl methylphosphonate monosodium salt, TRIMEPHONA, and 3-(trihydroxysilyl)propylamino-diphosphonate, TRIPADIPHOS] are multifunctional consolidants because they possess a self-condensable trihydroxysilyl [−Si(OH)_3_] moiety and phosphonate groups (one in TRIMEPHONA, two in TRIPADIPHOS). The conclusions drawn from this effort are outlined along the following points:

(1) Consolidants RC-70 and RC-90 exhibit rather low consolidation efficacies. This is not unexpected, as these are alkoxysilane-based and act simply as “fillers” for the pores of the gypsum.
(2) Consolidant TRIMEPHONA demonstrates an enhanced level of consolidation action. This is due to its double functionality, i.e. the presence of an anionic phosphorus-based moiety that anchors onto the gypsum surface, and a condensable silane triol unit.
(3) Consolidant TRIPADIPHOS shows excellent gypsum consolidation features and is much more efficient than all other tested consolidants. Its enhanced consolidation efficacy (per unit concentration) is assigned to its better gypsum anchoring ability.
(4) Based on the structural profiling of O–Ca–O angles present in the crystal structures of the model Ca–phosphonates Ca–C2D and Ca–C3D a bidentate chelating mode by a single TRIPADIPHOS molecule is proposed as the preferred grafting process onto gypsum.
(5) The phosphorus-based consolidants show a mild improvement in the compressive strength of gypsum.

## Supporting Information Available

Physicochemical characterization data for TRIPADIPHOS, characterization of treated gypsum samples, O–Ca–O angle values for all materials, cif files of Ca–C2D and Ca–C3D (PDF)
