# Supplementary material for: Profiling Novel, Multifunctional Silane-Phosphonate Consolidants for the Mitigation of Gypsum Stone Deterioration via Concerted Autocondensation/Surface Complexation Processes
Source: Cryst Growth Des. 2024 Jul 8;24(14):5959–73. doi: 10.1021/acs.cgd.4c00327 (PMC11261601; doi:10.1021/acs.cgd.4c00327)
Supplement: Supplementary file 1 — cg4c00327_si_001.pdf [file cg4c00327_si_001.pdf]

Supporting Information

For

Profiling novel, multifunctional silane-phosphonate consolidants for the mitigation of gypsum stone deterioration via concerted autocondensation/surface complexation processes

Stefania Liakaki-Stavropoulou,[a] *Argyri Moschona,*[a] *Ioannis E. Grammatikakis,*[a,†] *Duane Choquesillo-Lazarte,*[b] *and Konstantinos D. Demadis*[a,*]

[a] Crystal Engineering, Growth and Design Laboratory, Department of Chemistry, University of Crete, Voutes Campus, Heraklion, Crete, GR-71003, Greece

[b] Laboratorio de Estudios Cristalográficos, IACT, CSIC-Universidad de Granada, Granada-18100, Spain

* Corresponding author. E-mail: demadis@uoc.gr

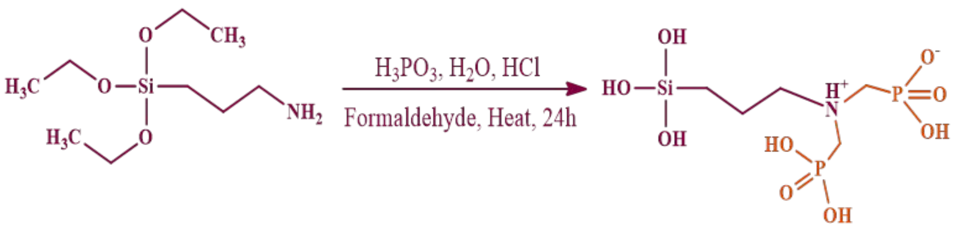

Fig. S1. Schematic representation of the Mannich-type synthetic strategy to prepare TRIPADIPHOS.

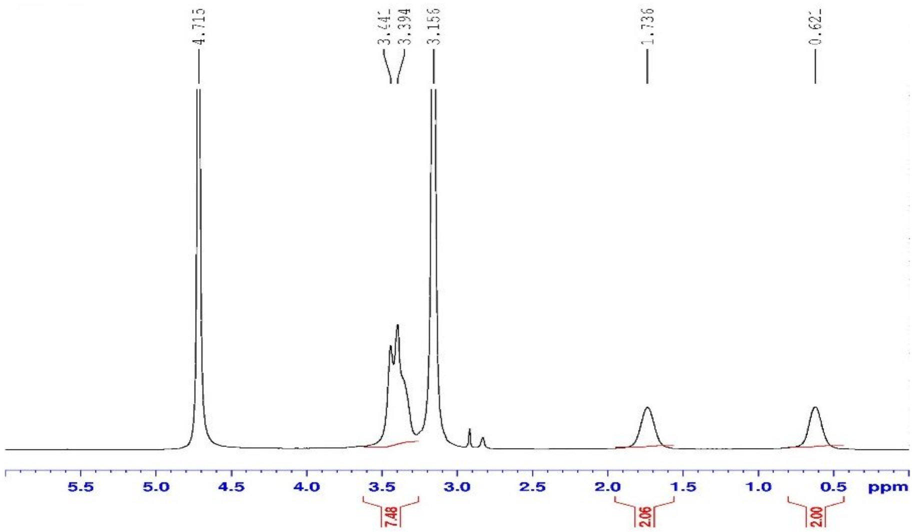

Fig. S2. ^{1}H NMR spectrum of TRIPADIPHOS in D_2O.

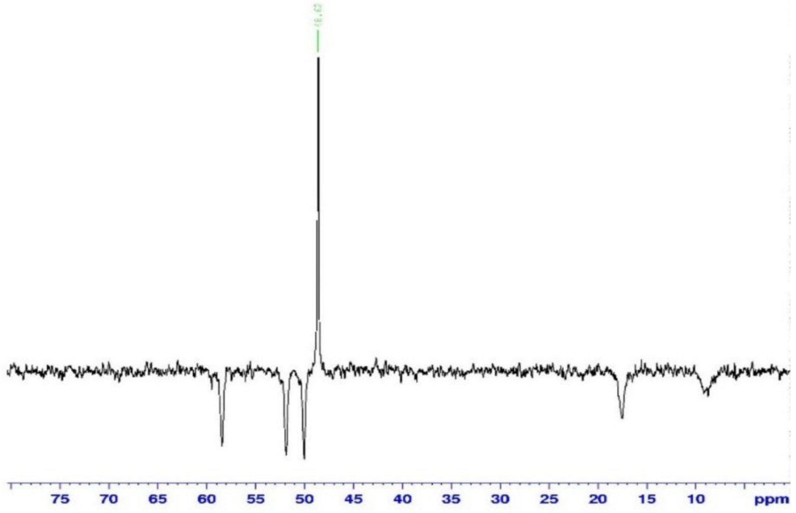

Fig. S3. ^{13}C NMR spectrum of TRIPADIPHOS in D_2O.

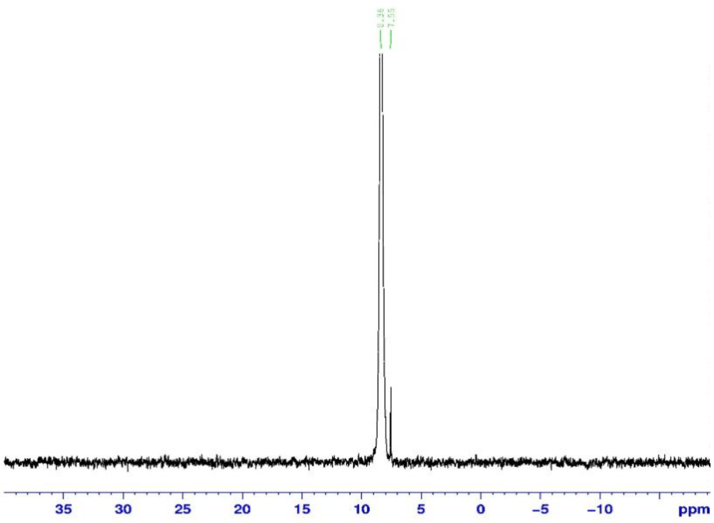

Fig. S4. ^{31}P NMR spectrum of TRIPADIPHOS in D_2O.

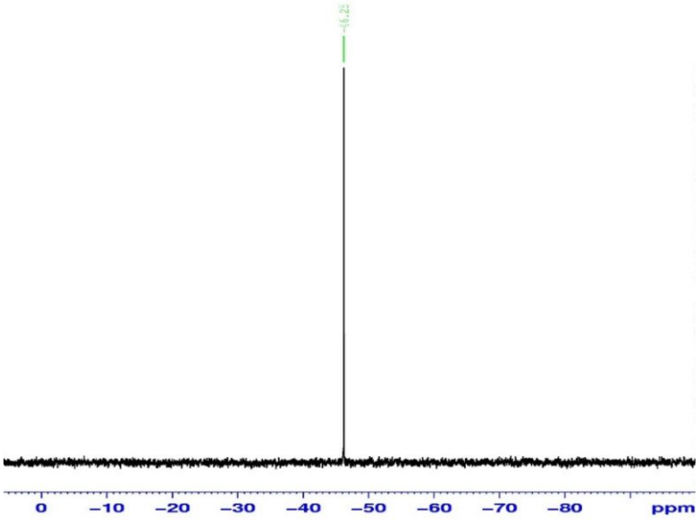

Fig. S5. ^{29}Si NMR spectrum of TRIPADIPHOS in D_2O.

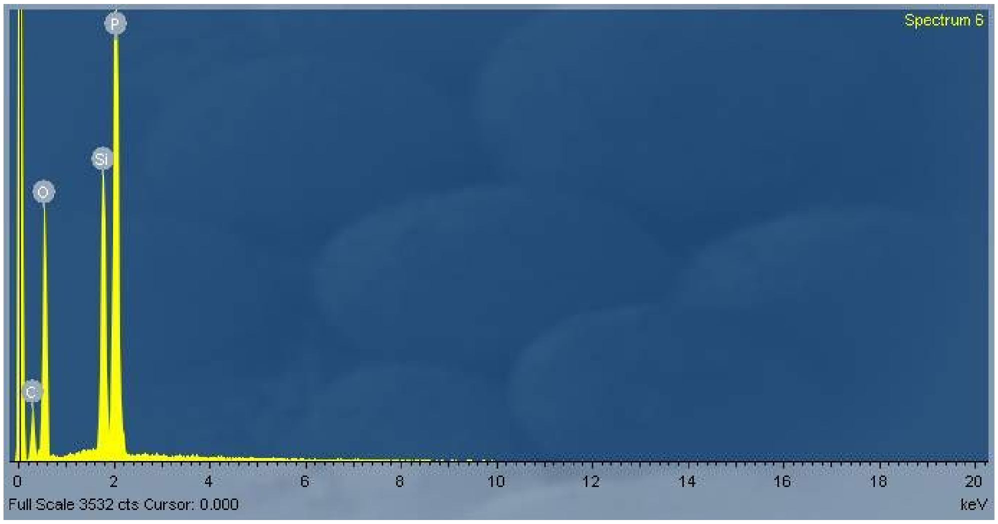

Fig. S6. EDS spectrum of solid TRIPADIPHOS.

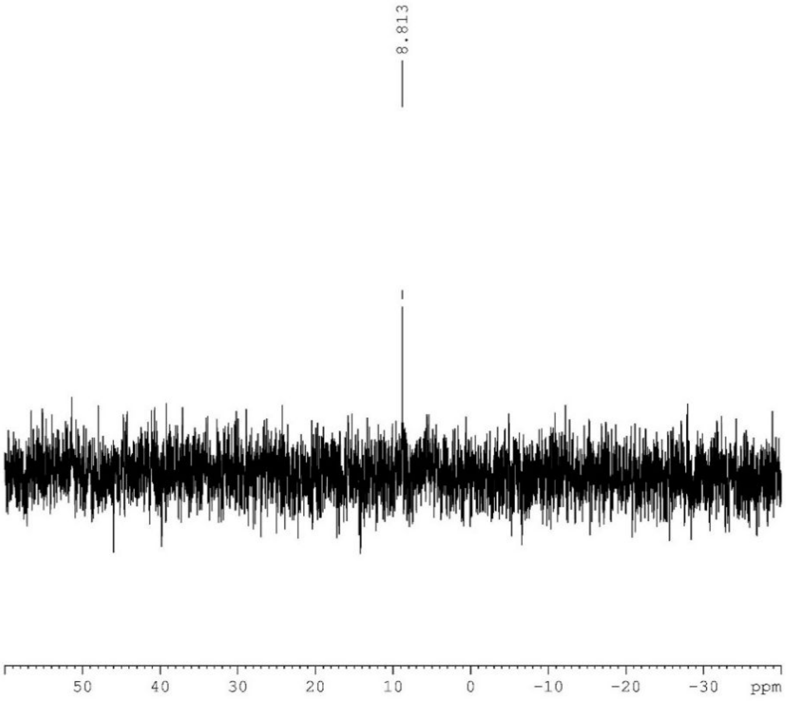

Fig. S7. ^{31}P NMR spectrum of acid-treated gypsum sample, consolidated with TRIPADIPHOS.

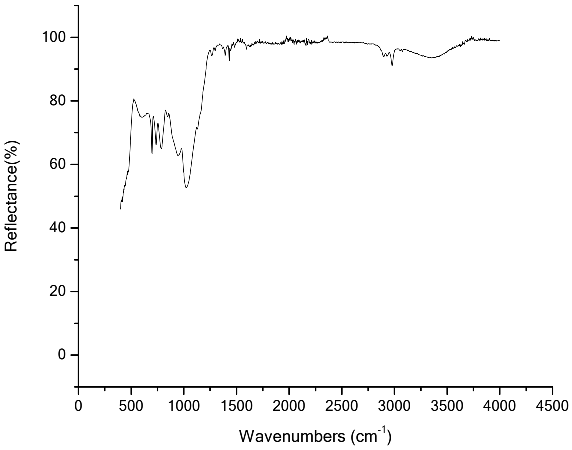

RC-90 (pure gel)

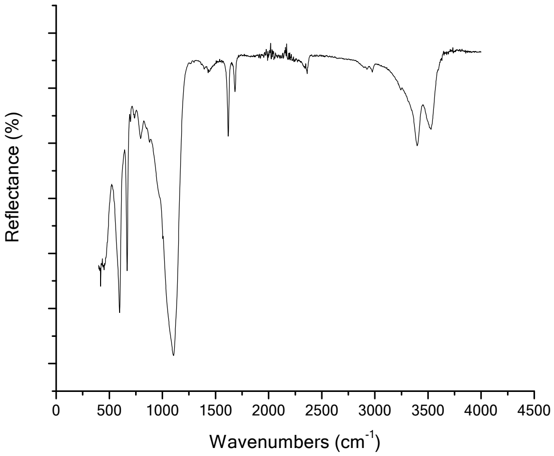

Consolidated gypsum with RC-90

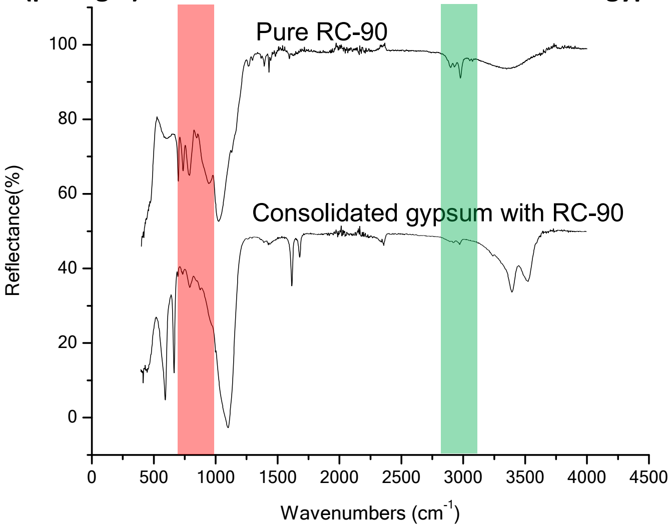

Comparison

Fig. S8. ATR-IR spectra of pure RC-90 after gelation (upper left) and gypsum treated with RC-90 (upper right), and their comparison (lower).

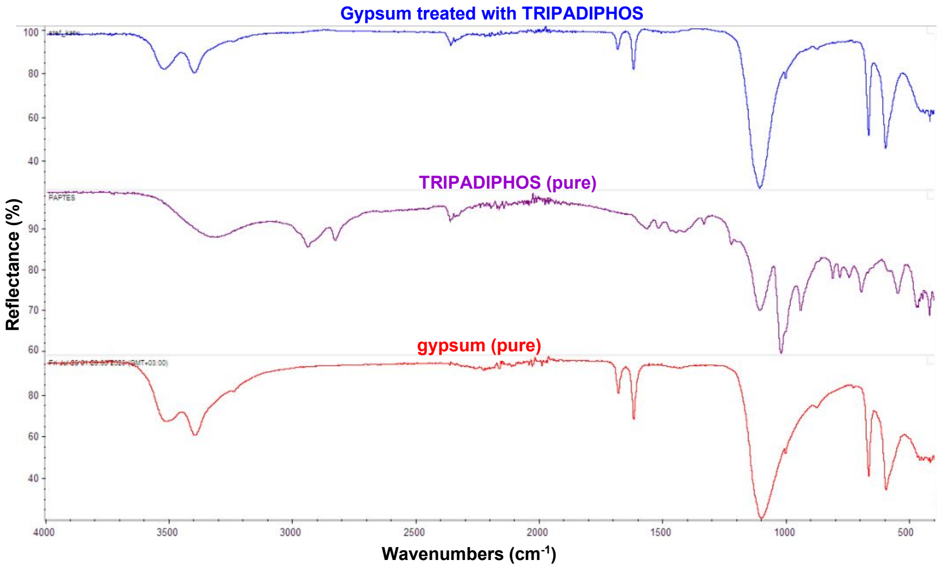

Fig. S9. ATR-IR spectra of pure gypsum (lower spectrum), TRIPADIPHOS (middle spectrum) and gypsum treated with TRIPADIPHOS (upper spectrum).

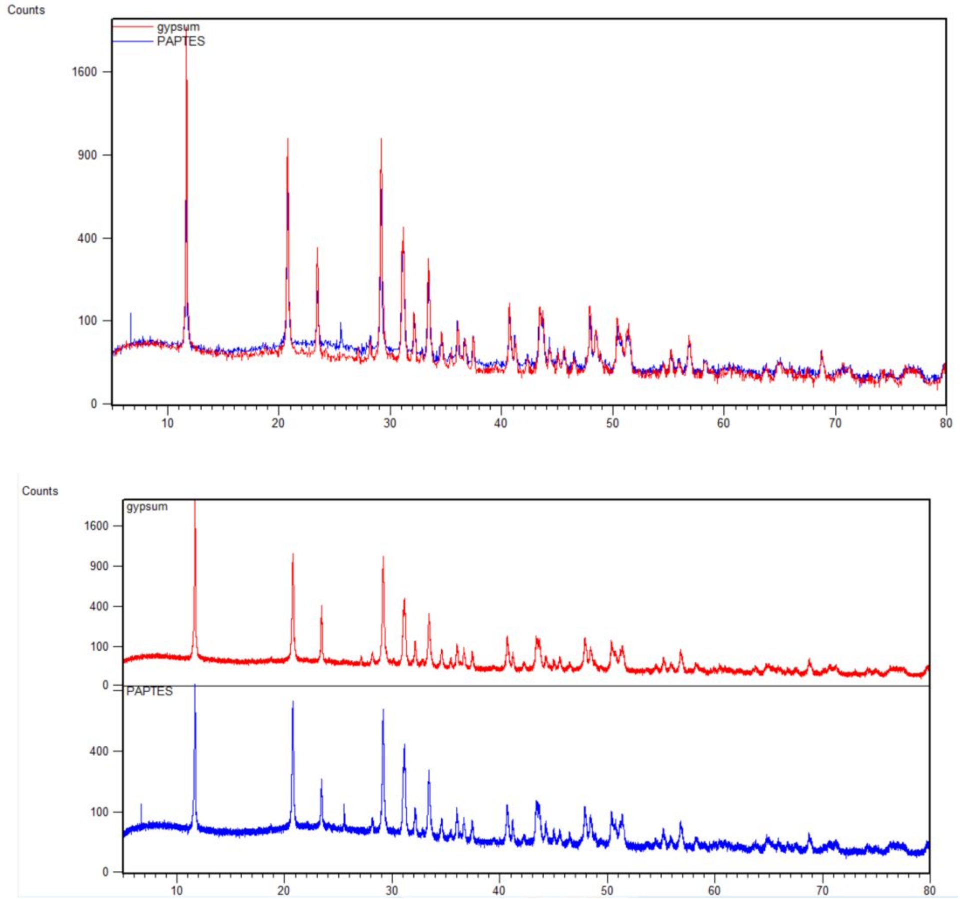

Fig. S10. XRD diffraction patterns of pure gypsum (red), and of gypsum treated with TRIPADIPHOS (blue).

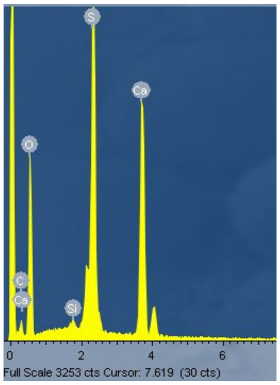

Element	App Conc.	Intensity Corrn.	Weight%	Weight% Sigma	Atomic%
C K	65.54	0.4710	36.86	0.77	49.99
O K	58.39	0.4076	37.94	0.77	38.63
S K	41.77	0.9890	11.19	0.24	5.68
Ca K	51.33	0.9707	14.01	0.27	5.69
Totals			100.00		

Fig. S11. EDS spectrum of pure gypsum and elemental analysis.

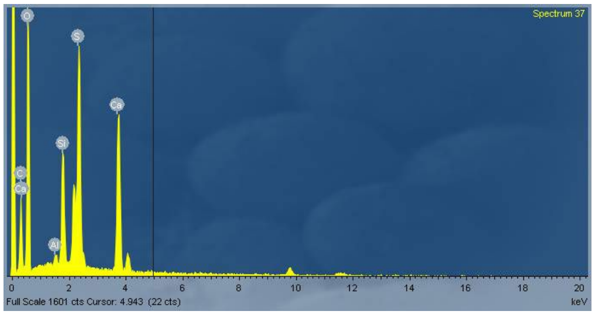

Element	App Conc.	Intensity Corrn.	Weight%	Weight% Sigma	Atomic%
C K	25.85	0.3650	14.46	0.68	22.00
O K	139.88	0.5296	53.93	0.61	61.59
Si K	8.15	0.9047	1.84	0.09	2.52
S K	54.82	0.9450	11.85	0.22	6.75
Ca K	74.36	0.9687	15.67	0.25	7.14
Totals			100.00		

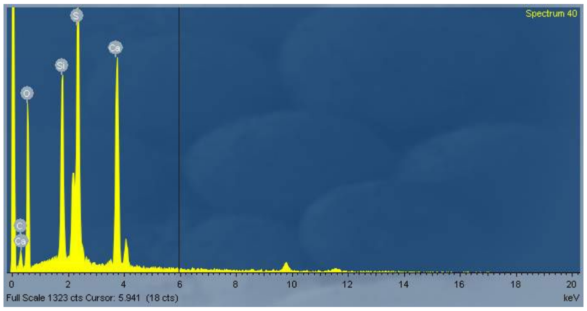

Element	App Conc.	Intensity Corrn.	Weight%	Weight% Sigma	Atomic%
C K	15.54	0.3030	13.79	0.85	22.81
O K	64.87	0.4099	42.54	0.77	52.84
Si K	3.86	0.9284	1.12	0.09	1.65
S K	66.49	0.9799	18.24	0.34	11.30
Ca K	82.53	0.9660	22.97	0.40	11.39
Totals			100.00		

Fig. S12. EDS spectrum of gypsum consolidated with RC-70 (upper) and RC-90 (lower) and elemental analysis.

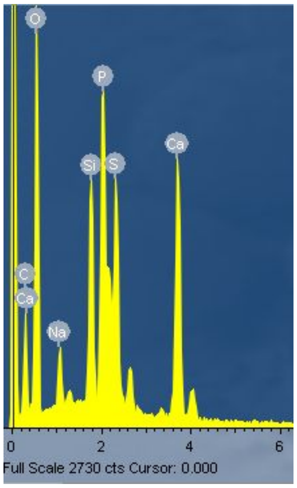

Element	App	Intensity	Weight%	Weight%	Atomic%
	Conc.	Corrn.		Sigma	
C K	25.85	0.3650	14.46	0.68	22.00
O K	139.88	0.5296	53.93	0.61	61.59
Si K	8.15	0.9047	1.84	0.09	1.20
P K	14.35	1.3060	2.24	0.13	1.32
S K	54.82	0.9450	11.85	0.22	6.75
Ca K	74.36	0.9687	15.67	0.25	7.14
Totals			100.00		

Fig. S13. EDS spectrum of gypsum consolidated with TRIMEPHONA and elemental analysis.

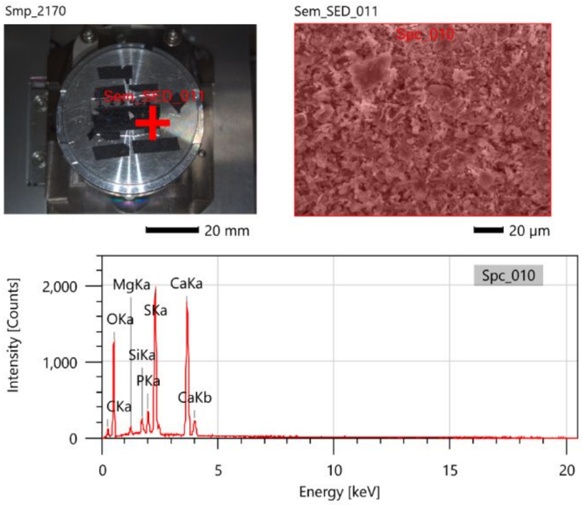

Signal SED
Landing Voltage 20.0 kV
WD 10.5 mm
Magnification x750
Vacuum Mode HighVacuum

Items	Value
measurement conditions	
Acceleration voltage	20.00 kV
Probe current	-
Magnification	x 750
Process time	T3
Measurement detector	First
Live time	60.00 seconds
Real time	60.47 seconds
Dead time	1.00 %
Count rate	1445.00 CPS

Display name	Standard data	Quantification method	Result Type
Spc_010	Standardless	ZAF	Metal

Element	Line	Mass%	Atom%
C	K	8.30±0.30	13.47±0.49
O	K	54.12±0.95	65.91±1.16
Mg	K	0.65±0.06	0.52±0.05
Si	K	1.03±0.06	0.72±0.04
P	K	2.19±0.09	1.37±0.05
S	K	13.33±0.19	8.10±0.12
Ca	K	20.37±0.30	9.90±0.14
Total		100.00	100.00
Spc_010			Fitting ratio 0.0201

Fig. S14. EDS spectrum of gypsum consolidated with TRIPADIPHOS and elemental analysis.

Table S1. All measured O-Ca-O acute angles in the structures of gypsum, Ca-C2D and Ca-C3D. Te Ca sites are color-coded.

O-Ca-O angle	Ca center	structure	material
98.40	Ca	gypsum	gypsum
92.87	Ca	Ca-C2D	Ca in Ca-C2D
99.05	Ca	Ca-C2D	
59.38	Ca1	Ca-C3D	Ca1 in Ca-C3D
84.82	Ca1	Ca-C3D	
77.86	Ca1	Ca-C3D	
87.70	Ca1	Ca-C3D	
81.02	Ca1	Ca-C3D	
95.21	Ca1	Ca-C3D	
78.34	Ca1	Ca-C3D	
80.21	Ca1	Ca-C3D	
76.39	Ca1	Ca-C3D	
75.03	Ca1	Ca-C3D	
76.35	Ca1	Ca-C3D	
89.29	Ca2	Ca-C3D	Ca2 in Ca-C3D
91.49	Ca2	Ca-C3D	
88.40	Ca2	Ca-C3D	
84.86	Ca3	Ca-C3D	Ca3 in Ca-C3D
83.02	Ca3	Ca-C3D	
58.12	Ca3	Ca-C3D	
80.38	Ca3	Ca-C3D	
86.97	Ca3	Ca-C3D	
82.87	Ca3	Ca-C3D	
79.20	Ca3	Ca-C3D	
74.29	Ca3	Ca-C3D	
80.98	Ca3	Ca-C3D	
77.37	Ca3	Ca-C3D	
86.77	Ca3	Ca-C3D	
82.53	Ca4	Ca-C3D	Ca4 in Ca-C3D
59.17	Ca4	Ca-C3D	
89.36	Ca4	Ca-C3D	
82.24	Ca4	Ca-C3D	
73.19	Ca4	Ca-C3D	

80.08	Ca4	Ca-C3D
73.77	Ca4	Ca-C3D
89.51	Ca4	Ca-C3D
82.50	Ca4	Ca-C3D
94.34	Ca4	Ca-C3D
99.62	Ca4	Ca-C3D
